# GrowScreen-Rhizo 3 - automated large-scale high throughput greenhouse phenotyping of plant root and shoot development

**DOI:** 10.1016/j.plaphe.2026.100213

**Published:** 2026-04-23

**Authors:** Laura Verena Junker-Frohn, Henning Lenz, Shiyan Jia, Alexander Putz, Jens Wilhelm, Constantin Eiteneuer, Sascha Adels, Olaf Mück, Anna Galinski, Jonas Lentz, Fabio Fiorani, Mark Müller-Linow, Kerstin A. Nagel

**Affiliations:** Institute of Bio and Geosciences, IBG-2: Plant Sciences, Forschungszentrum Jülich GmbH, 52425 Jülich, Germany

**Keywords:** Whole-plant phenotyping, Root phenotyping, Shoot phenotyping, Imaging, Rhizotron, Non-invasive

## Abstract

Roots play a pivotal role for plant performance, but they are difficult to access, which hampers quantitative measurements. Repeated imaging of rhizotrons, flat growth containers with a transparent side, has proven suitable to assess dynamics of root traits in indoor experiments. However, measuring hundreds of soil-grown plants with high temporal resolution remains a laborious challenge. We introduce a novel whole-plant phenotyping platform with a capacity of almost 900 rhizotrons, which we named GrowScreen-Rhizo 3. This platform was designed to image shoots and roots of individual plants simultaneously and derive digital proxy traits for biomass and growth. In addition, built-in weighing and watering stations deliver water use data for each rhizotron. To achieve the desired throughput (image all 896 plants once a day) a high degree of automatization and standardization was required. We realized a modular plant-to-sensor solution, using a fleet of automated guided vehicles (AGVs) to transport large rhizotrons (80 × 40 × 5 cm) to four measurement chambers for daily imaging, weighing, and watering. Simultaneous imaging of the root system with a high-resolution camera (116 μm per px) and the shoot from six different viewing angles allows to monitor plant growth with high spatial and temporal accuracy. First, we verified that moving plants to the measurement chambers did not significantly affect above- or belowground plant growth. Next, we measured phenotypic variation in root and shoot traits of 24 barley genotypes, parents of a nested association mapping population. Our analysis revealed that heritability of root traits such as root system depth and seminal root length was moderate to high (r^2^ = 0.52 and r^2^ = 0.93, respectively), enabling further assessment of increasing numbers of recombinant genotypes. The results demonstrate the suitability of GrowScreen-Rhizo 3 to phenotype a range of plant species characterized by various growth habits, including crop, niche, and wild plant species. We conclude that GrowScreen-Rhizo 3 will contribute significantly to the development of phenotyping pipelines for the identification of candidate genotypes with improved resource use efficiency and to pre-breeding processes of climate-resilient crops.

## 1 Introduction

Plant phenotyping is considered as one of the modern key tools for understanding growth and adaptation of plants to climate change and can accelerate breeding for climate-resilient crops [1]. High throughput phenotyping technologies in both controlled and field environments are required to overcome the phenotyping bottleneck [2]. Characterization of few hundred individual plants is necessary for QTL or association mapping and for closing the gap between genotyping and phenotyping [3]. In recent years, various high-throughput phenotyping technologies have been developed for precise and non-invasive plant phenotyping to meet the requirements of plant research and breeding [1,2,4].

Plant roots play a crucial role in water and nutrient supply for growth and photosynthesis, in anchorage in the soil, interaction with soil microorganism and have to sense and cope with a wide range of abiotic and biotic stresses etc. [[5], [6], [7], [8], [9]]. However, quantifying root traits of plants grown in field environments is still challenging due to the hidden nature of roots. Phenotyping root system architecture (RSA) traits in the field relies mostly on invasive and destructive methods, such as shovelomics or soil coring [10,11], or minimal invasive methods such as Minirhizotrons using imaging tubes in the soil [12], which only allow access to a limited part of the root system. Other methods applied in the field can only provide an indirect estimation about root system size or root distribution in the soil e.g. via measuring canopy temperature or spectral emission [4,13].

Although field-based methods are crucial, so far there are no methods for field application available to quantify growth and architecture of whole root systems over time. Furthermore, soil and climate conditions are highly variable in field experiments and even highly variable within one field, which is challenging e.g. for screening genotypes or studying stress effects. Therefore, combining approaches from field, laboratory, and greenhouse open new routes, especially for root phenotyping [2]. Root phenotyping under controlled environments allows measurements of root traits with high repeatability and low variation [14]. Additionally, there are several options for plant cultivation under controlled environments which enable a non-invasive observation of root development over time and simultaneous quantification of root and shoot traits. Increasing the capacity of lab- or greenhouse-based phenotyping approaches to high throughput gives the opportunity to phenotype whole plant populations and to speed up the genetic gain of selection of new elite germplasm within (pre)breeding processes [2,14].

The number of automated platforms for phenotyping root traits has increased in recent years. However, the number of high throughput platforms using soil-grown plants is still limited. Several soil-less methods appropriate for high throughput measurements of roots with a capacity of up to several hundred plants have been developed in the last years, using germination paper or cloths [[15], [16], [17], [18], [19], [20]], transparent media such as agar [21,22] or hydroponics or aeroponics [23]. Soil-less approaches have the advantage that the entire root system is accessible for imaging and repetitive measurements. However, growing plants in soil-less systems can affect root system architecture and development especially due to lacking soil structure or mechanical impedance. Most soil-less cultivation systems face the challenge in creating heterogeneity of water and nutrients and microbial communities unlike the ones that plants typically observe in natural soil. A compromise solution between soil-less and soil-based cultivation systems is the use of RhizoTubes, in which roots grow in transparent tubes filled with soil, but separated from the soil by a permeable membrane [24].

Non-destructive phenotyping of roots grown in opaque soil requires methods such as magnetic resonance imaging (MRI [25]) or computer tomography (CT [26]). Both methods allow analyzing root development in soil-filled pots or tubes in 3D, but typically with low to medium throughput, as the detection of thin structures such as roots requires high resolution and consequently longer measurement times [27,28]. For high throughput, optical sensors like RGB or monochrome cameras are more appropriate, as the acquisition time is negligible compared to MRI or CT measurements. However, non-invasive image-based quantification of root traits requires to grow plants in growth containers with at least one transparent side to make roots accessible for imaging. Plants could either be grown in transparent pots, columns or so-called rhizotrons which are growth containers with at least one transparent interface for non-destructive observations of root growth in soil [29,30]. Transparent side(s) must be covered to prevent visible light from reaching roots during plant development, e.g. by placing clear pots in opaque pots or shielding transparent sides with opaque plates or respective filters [[31], [32], [33]]. Dimensions of the growth container can determine the fraction of visible roots, i.e. larger diameter of round pots or columns or thicker rhizotrons generally can hide more roots in the soil. Visibility of roots can be increased by placing seeds at the transparent wall of clear pots [32] or by tilting rhizotrons with the transparent window facing downwards [31]. In recent years, different platforms for image-based root phenotyping of rhizotron grown plants have been developed. For raising the capacity, some platforms are based on thin rhizotrons with inner dimensions of a few millimeters, allowing to place many rhizotrons in a reasonable small cultivation area [[34], [35], [36]] or by placing several plants in one rhizotron [33,37]. However, limited space per plant has consequences for maximal experimental duration and the overlapping root systems of neighboring plants within a rhizotron make it difficult to extract root traits of individual plants. In the extreme example of the platform WinRoots, the 40 soy plants that are planted in one rhizotron with a spacing of 2.5 cm can only be phenotyped for up to 10 days [38].

The throughput of many published soil-based root phenotyping platforms is limited by human resources e.g. for imaging, but also for rhizotron or pot filling and plant irrigation. Especially low-cost platforms that require manual imaging with digital cameras often have a limited throughput [34,35]. To automate measurements, flatbed scanners could be permanently placed at the rhizotrons surface, but the total capacity of such a platform is limited by the availability of space and money [38]. To increase the capacity of platforms by automating processes, either sensors can be moved from plant to plant or plants can be transported to sensors. In the sensor-to-plant approach, rhizotrons have fixed positions and are typically aligned in rows and the camera or imaging cabinet is moved between the rows of rhizotrons either with automated guided vehicles (AGVs) (RootHTP [39]) or conveyor systems (GrowScreen-Rhizo 1 [31]). Phenotyping systems with fixed rhizotron positions as in GrowScreen-Rhizo 1 can be enlarged by extending the rows or by using several systems in parallel, which both limit the maximum capacity in terms of space, measurement duration, and costs. Plant-to-sensor approaches, in which plants are transported to fixed mounted sensors typically in measurement chambers, are more suitable for automated high throughput phenotyping. In recent years, platforms have been developed using gantry robotic systems within climate chambers (GLO-Bot, RootBot [40,36]). These platforms transport small, very thin rhizotrons automatically to an imaging station to assess specific traits, *i.e.* luminescence-based studies of root architecture [36,40]. For greenhouse applications, platforms based on conveyor belt systems have been developed to transport either groups of rhizotrons as in the medium throughput platform GrowScreen-Rhizo 2 [41] or custom designed rhizo-pots [33] to an imaging station. The rhizo-pots are designed with a tilted front side and a height of 40 cm to match the carrier size used in a high throughput platform originally developed for shoot phenotyping [42]. So far, no non-commercial platform is available that combines large rhizotrons with automated imaging for high throughput phenotyping of hundreds of plants.

To close these gaps, we developed the automated high throughput phenotyping platform GrowScreen-Rhizo 3 based on the experience of the previous platforms GrowScreen-Rhizo 1 and 2 [31,41]. GrowScreen-Rhizo 1 consisted of 72 rhizotrons with fixed positions and a measurement chamber moving along the rhizotrons positions for imaging [31]. For increasing flexibility and modularity, in the successor platform GrowScreen-Rhizo 2 with a capacity of 80 plants, rhizotrons were moved in groups on a conveyor belt into the stationary measurement chamber [41]. In the current version GrowScreen-Rhizo 3, we further increased the flexibility and scalability by transporting the rhizotrons within the platform using automated guided vehicles (AGVs) instead of relying on fixed conveyor systems. The new concept allowed us to increase the capacity to in total 896 rhizotrons. While most platforms either focus on shoots or roots, GrowScreen-Rhizo 3 (as the previous versions) enables simultaneous phenotyping of shoot and root traits non-invasively. The platform design is modular with measurement chambers and AGVs working in parallel enabling daily measurements of all plants and allowing high flexibility in experimental designs and adjustments to meet specific requirements. The platform includes a semi-automated filling line for standardized filling of rhizotrons. The aim of this publication is to present the features and workflow of the novel platform and to demonstrate its potential to be used for a wide range of crop, niche, and wild plant species. For validation, we tested the effect of daily movement and daily imaging on root and shoot development of different plant species. In addition, we showed the potential of the novel platform by whole-plant phenotyping of 24 barley genotypes.

## 2 Material and methods

### 2.1 Overview of the platform

**Fig. 1:**
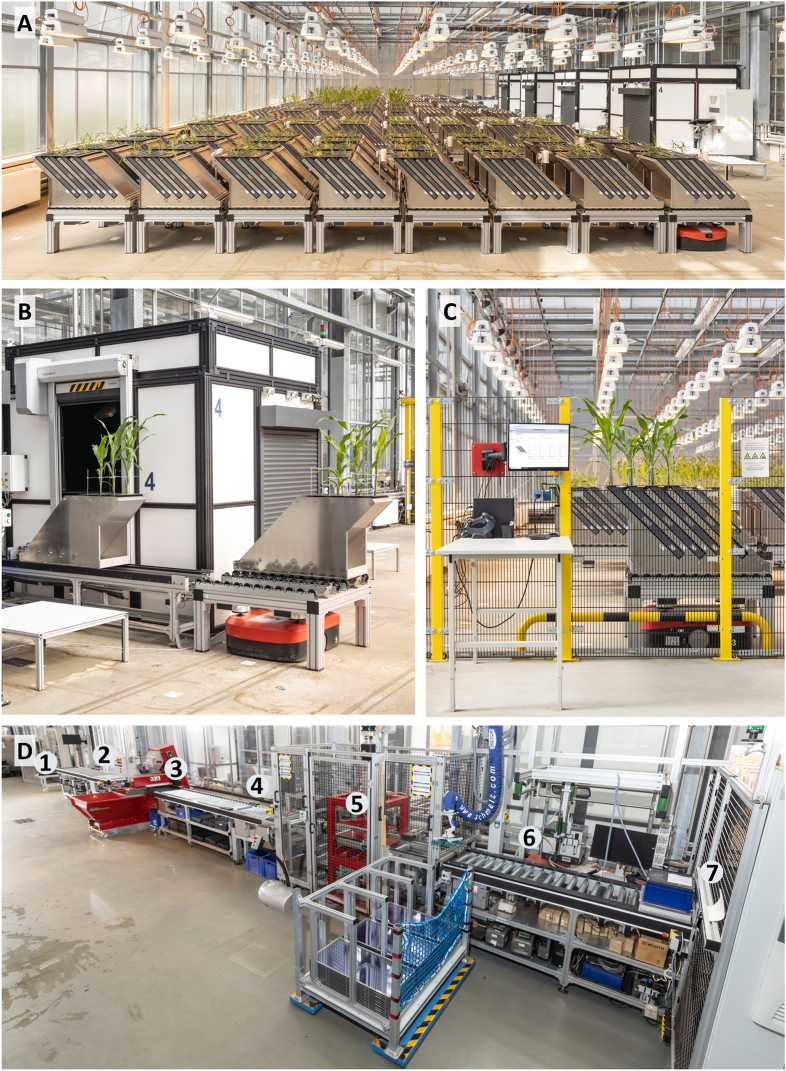
GrowScreen-Rhizo 3 platform. A) Overview of the greenhouse compartment with 112 tables in the plant cultivation area in the center and four measurement chambers at the right side. One of the five red AGVs is visible below the front right table. B) Measurement chamber with a table in front, one magazine remaining on the table waiting for imaging and the second magazine on the conveyor in front of the chamber with one rhizotron pulled into the chamber. C) One of three presentation stations for sowing, manual measurements, and shoot harvest. D) Semi-automated filling line for rhizotron filling and root harvest. The labels in subfigure D mark the different stations of the filling line and are explained in the Material and Methods.

The GrowScreen-Rhizo 3 platform is in a greenhouse of the Institute of Bio- and Geosciences, IBG-2: Plant Sciences at Forschungszentrum Jülich GmbH (50°54′36.4″N 6°24′45.7″E). The phenotyping platform encompasses a greenhouse area of around 640 m^2^ and features a modular plant-to-sensor approach (Fig. 1A, S1A). The design of the platform was developed in cooperation between the institute IBG-2: Plant Sciences and the company Maschinenbau Kitz GmbH (Troisdorf, Germany). Plants are grown in soil-filled rhizotrons, which are arranged in groups of eight on tables, arranged in two magazines with four rhizotrons each (Fig. 1A–C, S1A,B). The platform consists of a large plant cultivation area (Fig. 1A), four measurement chambers for automated root and shoot imaging (Fig. 1B), three presentation stations for sowing, manual measurements, and shoot harvests (Fig. 1C, Fig. S1A) and a semi-automated filling line for filling rhizotrons (Fig. 1D). Tables are transported within the platform via five automated guided vehicles (AGVs; Grenzebach Maschinenbau GmbH, Asbach-Bäumenheim/Hamlar, Germany), which can drive under each table (Fig. 1A–C), lift it slightly, and then move it along a grid system of markers permanently fixed to the floor. The AGVs are controlled by a fleet manager software, which optimizes pathways for tasks such as transport of tables from the cultivation area to the pre-defined measurement chamber and temporarily reserves this path to avoid crashes with other AGVs (Fig. S2). Plants are moved with maximum speed of 1 m s^−1^ (equals walking speed), with gentle acceleration and deceleration with maximum 0.5 m s^−2^. The whole platform is fenced to fulfil work safety requirements.

### 2.2 Rhizotrons

The rhizotrons measure 80 cm in height, 40 cm in width and 5 cm in thickness, with inner dimensions of 75.7 x 36.0 × 2.4 cm and a filling volume of 6.42 l. The rhizotrons were developed in cooperation with the company Happ Kunststoffspritzgusswerk und Formenbau GmbH (Ruppichteroth, Germany). The rhizotron body (Fig. S1C) is made of a grey durable plastic (PC-ABS) using injection molding, featuring a honeycomb structure to ensure lightweight, but stable construction (2785 ± 2 g). For automated watering, which is done inside the measurement chambers, the rhizotron body has four small openings near the top, right above the soil surface (Fig. S1C). The rhizotron bodies are complemented with 1 cm thick transparent plates made of polycarbonate (4648 ± 11 g). The plates are stabilized with a 1 cm wide metal frame (stainless steel) and are screwed to exchangeable cage nuts in the rhizotron bodies. Rhizotrons are kept at an angle of 45° in the magazines and in the measurement chambers, with the transparent plate facing downwards (Fig. S1B). Eight rhizotrons are grouped in two magazines per table, and can be individually accessed for imaging, filling, and harvest by first pulling the respective magazine from the table on a skate wheel conveyor (Fig. 1B) and then pulling the respective rhizotron out of the magazines (Fig. S1B). Both magazines and rhizotrons are placed on roller tracks to ensure smooth movements. Each rhizotron body and plate is labelled with an individual barcode, which are scanned at all positions, where individual rhizotrons are handled (imaging, filling, harvesting, and storing). After sowing, black adapters (44 × 16 cm, made of aluminum composite plates) are placed on the rhizotrons (Fig. S1B). They keep the substrate surface exposed but protrude around the rhizotron edges to match the measurement chambers’ separator opening. Thereby, they provide a homogenous background for shoot imaging and prevent damage to low-hanging leaves (Fig. S3A). For climbing plant species or plants with unstable shoots such as lentil, shoots are supported by transparent retainers to ensure reliable representation of plants on shoot images (Fig. 3F). The total weight of a rhizotron filled with well-watered peat substrate (65% gravimetric water content; GWC) is approx. 11 kg.

### 2.3 Cultivation area

The cultivation area covers 300 m^2^ of a large greenhouse compartment (Fig. 1A, S1A, S2). 112 tables with a total number of 896 rhizotrons are arranged in 7 double rows with 16 tables per double row. Air temperature and relative air humidity are regulated using ventilation, shading, evaporative cooling, and fan convectors for heating and cooling. Additional illumination for the plants is provided by 136 assimilation lamps (MGR-K 400-SON, DH Licht, Wuelfrath, Germany) equally distributed over the plant cultivation area. Lamps are switched on between 6:00 and 22:00, whenever natural irradiation measured outside the greenhouse is below 330 W m^−2^. Every second table is equipped with sensors recording 10-min averages for air temperature, relative air humidity, and light intensity in a central microclimate database for monitoring of spatial and temporal changes in environmental conditions. To facilitate access to the plants for sowing, plant inspection, manual measurements, and shoot harvest even during operation, tables can be requested at three presentation stations, where access to four rhizotrons at a time is granted through a cutout in the fence (Fig. S1A). A computer with customized user-guided software provides information about the requested plants positioned upfront and the required measures, e.g. which genotype should be planted, or which shoot to be measured or harvested.

### 2.4 Measurement chambers

**Fig. 2:**
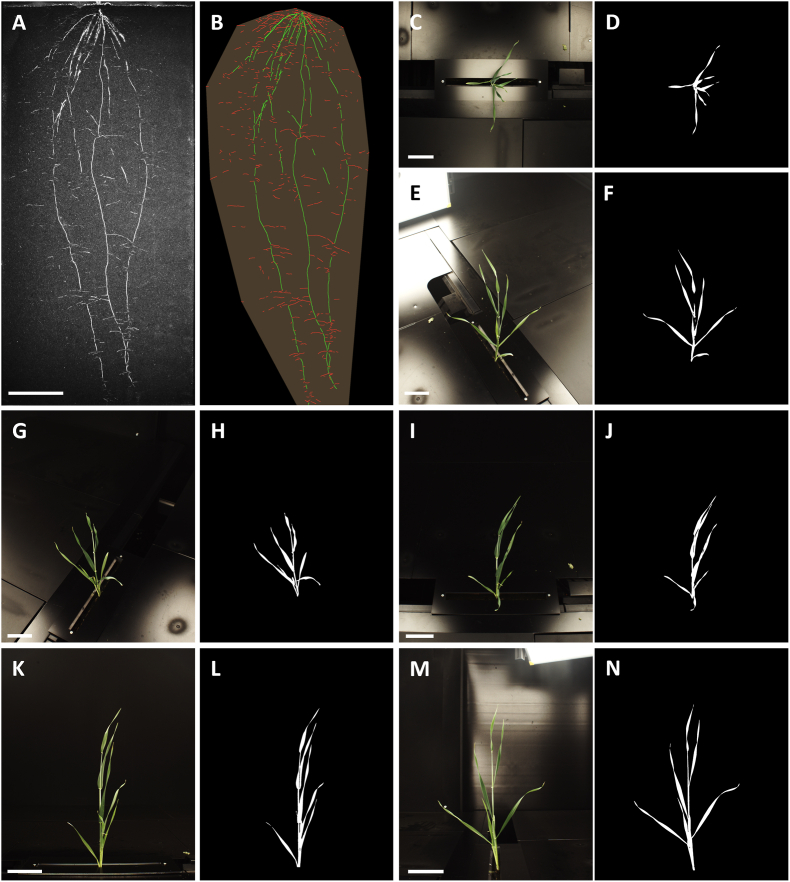
Images and masks of a representative barley plant 24 days after transplantation from all camera positions. Monochromatic root image (A) and analyzed color-coded image with main roots labelled in green and lateral roots in red (B). Shoot top view image (C) and segmented mask (D). Three shoot 45° downwards views arranged in 120° angles to each other (E, G, I) and respective segmented masks (F, H, J). Two shoot side views arranged in 90° angles to each other (K, M) and respective segmented masks (L, N). Scale bars indicate 10 cm.

Measurement chambers are sized 2.8 by 2.5 m with a height of 3.0 m (Maschinenbau Kitz GmbH; Fig. 1D and A). All measurement chambers are equally equipped for whole-plant phenotyping with one root and six shoot cameras, illumination panels for imaging, load cells for weighing, an irrigation system for watering, and a high-speed-door (ASSA ABLOY Entrance Systems Germany GmbH, Lippstadt, Germany; Fig. S3). Tables are transported to a conveyor belt in front of the measurement chambers. One magazine at a time is positioned on the conveyor belt and transported to the open door of the measurement chamber (Fig. 1B). Individual rhizotrons are then pulled from the magazine into the measurement chamber for imaging, weighing, and watering. During imaging, the rolling shutter gate is closed to ensure constant illumination of the plants. The transparent rhizotron side is imaged completely with a high-resolution monochrome camera with a resolution of 116 μm per pixel (29 MP AVT GT6600B, Allied Vision Technologies GmbH, Stadtroda, Germany, combined with a Zeiss Distagon T 2,0/35 ZF-I objective, Carl Zeiss AG, Oberkochen, Germany). The camera's optical axis points orthogonally towards the rhizotron's surface from 85 cm (Fig 2A and Fig. S3A,B). The rhizotron is illuminated by two LED bars (EFFI-Flex-40-00-SD-P2, Rauscher GmbH, Olching, Germany; Fig. S1D), which are positioned along the left and right sides of the rhizotron. Plant shoots are imaged with six cameras (5.1 MP AVT Mako G-507C POE camera, Allied Vision Technologies GmbH combined with Fujinon HF12XA-5M objective, FUJIFILM Europe GmbH, Ratingen, Germany; Fig. S3A and B)., which are positioned at a distance of about 100 cm from the shoot (Fig. S3A and B). One camera takes a top view of the shoot (Fig. 2C), three cameras are placed around the shoot at an angular separation of 120°, pointing down at an angle of 45° (Fig. 2E,G,I), and two cameras are mounted orthogonal to the vertical shoot axis with an angular separation of 90° (Fig. 2K,M). Each shoot camera is embedded in an illumination panel (EFFI-FD-500-500-000-50-1M12P, Effilux GmbH, Munich, Germany; Fig. S3A and B) to optimize illumination. Four load cells (SWB505 MM CS 30 kg C6 EN1090, Mettler-Toledo GmbH, Gieβen, Germany) enable precise weighing of the rhizotrons (resolution ±1 g). An in-house developed irrigation system backed by a peristaltic pump (Heidolph Hei-FLOW Precision, Heidolph Scientific Products GmbH, Schwabach, Germany) is used to supply plants with water or nutrient solution, either with a fixed volume or based on a target weight for each individual rhizotron to maintain the soil water content (Fig. S3C). For this purpose, four tubes are automatically inserted into the four openings near the rhizotron's top to irrigate plants with up to 100 ml water or nutrient solution at a time (Fig. S1C). All processes, imaging, weighing, and watering, are fully automated and monitored using unique rhizotron IDs.

**Fig. 3:**
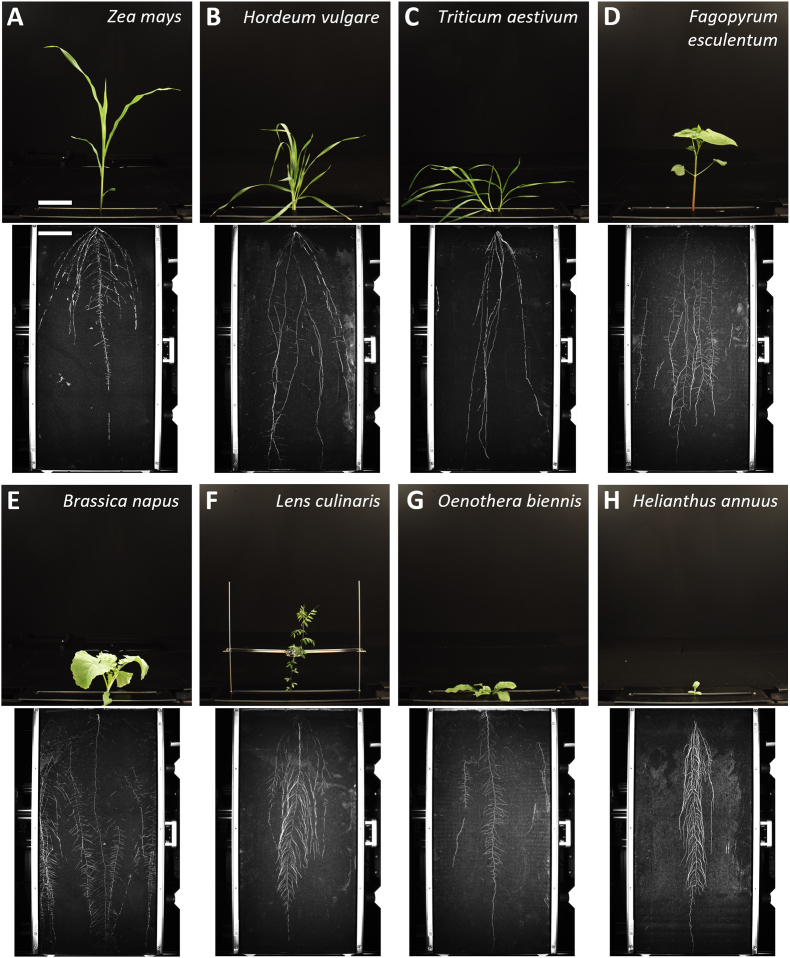
Representative images of different plant species grown in GrowScreen-Rhizo 3. A) Maize (15 DAT); B) barley (22 DAT); C) wheat (26 DAT); D) buckwheat (22 DAT); E) rapeseed (25 DAT); F) lentil (26 DAT); G) common evening primrose (41 DAT); H) sunflower (13 DAT). Scale bars indicate 10 cm.

### 2.5 Rhizotron filling line

The filling line for semi-automated filling of rhizotrons (Maschinenbau Kitz GmbH) is 25 m long and covers an area of approx. 65 m^2^ outside of the greenhouse compartment (Fig. 1D). In a semi-automated process, 2-3 persons can fill about 25 rhizotrons per hour with a standardized amount and compression of substrate. Tables with empty rhizotron bodies are delivered by the AGVs to the filling line input (1). One magazine at a time is pulled through an automated high-speed door and its four empty rhizotron bodies are transferred manually onto a conveyor belt (2). In-house developed filling frames (height 6 cm) are tightly placed onto the rhizotron bodies prior to filling. All four rhizotron bodies are automatically transported through a tray filler (Da Ros RC7-TR31, Da Ros, Sarmede, Italy; 3). The tray filler settings are adjusted to soil type and moisture content, to ensure comparable dry mass of substrate also in drought experiments. Typically, 3.3 kg of peat substrate with 65% GWC corresponding to a soil water potential of about −0.2 MPa are used per rhizotron. Afterwards, surplus substrate on the frames is manually cleaned and filled rhizotron bodies are automatically transported with frames to the weighing station (4), where the amount of filled substrate is confirmed, before the substrate is compacted using an automated hydraulic press (5). The pressing depth is adjusted to the respective soil type and moisture content. For rhizotrons of the presented barley experiment a substrate filling weight of 3370 ± 62 g was set, resulting in a substrate density of 0.52 ± 0.01 kg L^−1^. After manually removing the frames, transparent plates are lifted from an ergonomic lifting device on the filled rhizotron bodies using a vacuum tube lifter (J. Schmalz GmbH, Glatten, Germany) and screwed to the rhizotron bodies using a stationary screwdriver (MINIMAT 347-428U, Deprag Schulz GmbH, Amberg, Germany; 6). Finally, complete rhizotrons are automatically transported to the end of the filling line (filling line output; 7) and pushed at an angle of 45° with the transparent plate facing downwards into their respective slot of a magazine. The magazine is the same one, from which the empty rhizotron bodies was taken at the filling line input (1), and which was meanwhile automatically transported on its table to the filling line output (7) by an AGV. Once all the rhizotrons of one or both magazines on a table are filled, the table is automatically transported back to the cultivation area by the AGV. At the end of an experiment, the filling line is utilized in reverse direction for root harvest and substrate disposal. Tables are transported to the filling line output (7) and rhizotrons are individually taken from magazines for disassembly (6). Transparent plates are unscrewed, roots harvested or soil disposed of, and rhizotron bodies and plates are cleaned. Finally, empty bodies are placed for storage reasons into magazines at the filling line input (1).

### 2.6 Experimental procedures

GrowScreen-Rhizo 3 experiments are setup in a central plant database, that contains meta data for each rhizotron, including filling, genotype, imaging and harvest information. All experimental plants are assigned to groups based on substrate type, treatment, genotype, and harvest date, to address these groups individually for frequency of watering, imaging, manual measurements, shoot and root harvest, and to apply different imaging routines and treatments if needed. For each experiment, a suitable table arrangement in the cultivation area is selected and plants are automatically or manually distributed across tables, to match the desired experimental design.

As first practical step in the experimental workflow (Fig. S4), substrate is shredded from big bales (Peat loader CT2, Da Ros) and mixed (MX1000, customized, Da Ros). As a standard, fine peat substrate is used, but other substrates or soils can be used, too. Water and nutrient content of the substrate can be adjusted for example by spraying water or by adding fertilizer in the substrate during mixing. After filling the rhizotrons with the prepared substrate, the interim target weight of each rhizotron is determined in the measurement chambers. Prior to the start of experiments, rhizotrons can be watered daily to the interim target weight, to avoid drying of the upper substrate layer and ensure comparable soil water content between all rhizotrons of an experiment regardless of day of filling (in case rhizotrons are filled over several days).

Sowing seeds or transplanting of pre-germinated seedlings takes place at the presentation stations. Tables are delivered to the presentation stations and users are guided by on-screen instructions. Plant establishment is facilitated by initial watering of 100 ml to moisten the upper substrate layer. After sowing or transplanting, the adapters are placed on top of the rhizotrons, tables move to the cultivation area, and the experiment is initialized by automated weighing, and imaging in the measurement chambers. The determined weight can be used as target weight for watering throughout the experiment to keep soil moisture content in rhizotrons constant. When not being measured, plants are positioned at a defined position in the cultivation area. Throughout the experiment, plants are automatically imaged and irrigated in a user-defined frequency, which are typically daily measurements at the same time of day. Including watering with up to 100 ml, measurements take about 12 min per table, *i.e.* 1:30 min per rhizotron.

At harvest, the first step is typically sampling of the shoots, which take place at the presentation stations. Plants are delivered in groups by the AGVs, and on-screen instructions aid fast and reliable shoot sampling. For each harvested plant, a barcode to label the samples is printed. After cutting the shoots, adapters are removed. For root harvest, tables are delivered to the filling line output, where rhizotrons are disassembled by unscrewing the transparent plates. For root sampling, labels for root samples are printed and roots are washed, otherwise, substrate is discarded from the rhizotrons bodies.

For semi-automated tasks such as rhizotron filling, sowing, and harvesting, users are guided by customized software for an easy and intuitive working experience that helps to reduce human errors. The software layers controlling the processes during an experiment are summarized in Fig. S5.

### 2.7 Image analysis

The time series of root images are analyzed using the customized software GROWSCREEN-Root and the batch analysis routine as described in [31]. The labelled root structures are used to quantify the root traits such as planar root length of main and lateral roots, root system depth, root system width, and convex hull area (Fig. 2B, for trait definition see Table S1).

The shoot images from all six cameras are independently analyzed in a two-step process. First, images are semantically segmented into the plant object and background via an artificial neural network. The segmentation information from the six different view angles is then used to predict leaf area and plant height.

Shoot image segmentation is performed using the U-Net, a fast fully convolutional neural network specifically designed for semantic segmentation tasks [43]. Python (version 3.8.10) was used to train a PyTorch-based (version 2.0.1 [44]) U-Net implementation from an open-source segmentation model repository [45] using a timm-EfficientNet-b6 encoder and pre-trained weights from ImageNet [46] data as a starting point. The network was trained with 1500 manually annotated ground-truth image data, including 500 images each of barley, maize, and sunflower plants at different growth stages, respectively. The high-resolution input images with a size of 2464 (W) x 2056 (H) pixels were tiled according to an 8 x 7 grid of standard size 512 x 512 pixel. For stitching during interference, tiles were padded with a 5-pixel border with zero values, to avoid stitching artefacts when merging tiles by keeping the maximum predicted value for overlapping pixels, thus excluding the padded border in the final image. Training was performed iteratively, with data being randomly shuffled and split into 80% training and 20% validation data. Loss was calculated via Soft Binary Cross Entropy and optimization done using Adam [47] with an initial learning rate of 0.001.

Post-processing of predicted segmentations was performed using standard image processing techniques from the python libraries OpenCV [48] and Scikit-image [49]. Pixel predictions with a probability lower than 85% were not considered as plant pixels. Connected objects smaller than 50 pixels were removed from the prediction to reduce noise from image artefacts, caused, for example, by moss growth on the soil surface or other artefacts on the rhizotron body. Holes smaller than 50 pixels inside predicted plant structures were closed as well. Additionally, it was checked whether a plant shoot was visible at all in the sowing area. If not, other objects wrongly classified as plant parts were removed. Likewise, since only a single loosely connected pixel cluster representing the plant shoot was expected, objects with more than 100 pixels from the largest cluster near the sowing area were considered false positives and removed. Final validation of segmentation and post-processing using standard metrics (F1, precision, recall) was performed with a test image set of 100 annotated images of each species, revealing a high performance for all three plant species indicated by a mean F1-score of 0.9647 ± 0.1355. Barley performs best with an F1 score of 0.9846 ± 0.0061, maize very close with 0.9836 ± 0.0217 and sunflower a little worse with 0.9153 ± 0.2471.

Based on the semantically segmented image masks the predicted leaf area was predicted via Gaussian process regression, which is widely used in supervised learning and which makes predictions from prior knowledge via kernel functions [50]. The reference data were collected in two independent correlation experiments with barley grown under controlled conditions, in which the height and leaf area of the plants was determined at different growth stages. For computing predicted leaf area of a plant, the total number of predicted plant pixels for each of the six segmented masks was passed together with the camera type (top, 45°, side) as the input vector to the regression model. Combining a linear and rational quadratic kernel and using a constant mean function worked best in this scenario. Model performance was evaluated running 25 iterations of 5-fold cross validation that yields an average mean absolute percentage error (MAPE) of 0.0774 ± 0.0119.

Height of barley plants was derived from the pixel height of semantic segmentations of the two side camera images. The averaged maximum pixel height displayed a strong linear relationship with the manually measured ground truth height data. A linear fit of the data resulted in a MAPE of 0.1678, r^2^ = 0.9811 and a conversion factor of 0.0263 cm per pixel.

### 2.8 Experimental settings

For all experiments, greenhouse settings were 22 °C/18 °C day/night temperature, 55% relative humidity, with additional illumination from 6 a.m. to 10 p.m. The standard peat substrate (Sondermischung Mini Tray, Balster Einheitserdewerk GmbH, Fröndenberg, Germany) with 65% GWC was used. Seeds were pre-germinated in climate chambers in darkness between wet filter paper: wheat and barley for 24 h at 20 °C/18 °C (16h day), maize for 48 h at 20 °C/18 °C (16h day), sunflower for 30 h at 22 °C. Seeds were transplanted when root development just started, *i.e.* visible root tips were about 1-2 mm for wheat, barley, and sunflower and 5 mm for maize. Rhizotrons were imaged daily and watered to target weight.

Experiments were ended when the roots of the first genotypes or treatments reach the bottom of the rhizotrons. At harvest, shoots and roots are sampled. Shoots are cut and fresh weight is weighed immediately. Leaf area of harvested plants was measured destructively using a leaf area meter (LI-3100, LI-COR® Biosciences, Lincoln, NE, USA). Shoot dry weight was determined after a minimum of three days of drying at 60 °C. Roots were coarsely washed by hand and stored in distilled water, followed by fine washing and storage in 50% ethanol until they were scanned (Expression 12000XL scanner, 600 dpi; Seiko Epson Corporation, Suwa, Japan). Scanned root length was determined using WinRhizo Regular 2017 commercial software using an empirically set grey thresholding value.

### 2.9 Experiments

First, we tested the suitability of GrowScreen-Rhizo 3 for a range of monocotyledonous and dicotyledonous plant species. We grew maize (*Zea mays* ‘Gelber Badischer Land’), barley (*Hordeum vulgare* ‘Kombyne’), wheat (*Triticum aestivum* ‘Julius’), buckwheat (*Fagopyrum esculentum* ‘Eskalar’), rapeseed (*Brassica napus* ‘Ability’), lentil (*Lens culinaris* ‘Gudo’), evening primrose (*Oenothera biennis*), and sunflower *(Helianthus annuus* ‘AB-OR-8’) plants for two to six weeks in rhizotrons, depending on the plant species. N = 8 replicates were used per plant species.

To confirm that plant movement does not affect root and shoot traits, two movement experiments were conducted. In the first experiment starting 2022-02-04, N = 8 maize (*Zea mays* ‘Gelber Badischer Land’, Rolf Späth, Rastatt) and wheat (*Triticum aestivum* ‘Julius’, KWS Lochow GmbH, Bergen, Germany) were grown for 25 days after transplanting (DAT). For the second experiment starting date 2023-05-12, sunflower (*Helianthus annuus* ‘AB-OR-8’, NS Seme, Novi Sad, Serbia) was grown for 13 DAT (N = 8). Three handling protocols were compared: 1) plants that were not moved for the duration of the experiment (not moved + not imaged), 2) plants that were moved daily by AGVs, but without pulling the rhizotrons into the measurement chamber and therefore without imaging (moved + not imaged), and 3) plants that were moved and imaged daily (moved + imaged).

To develop the pipeline for shoot image analysis, a correlation experiment with maize (*Zea mays* ‘Gelber Badischer Land’), barley (*Hordeum vulgare* ‘Barke’, Saatzucht Josef Breun GmbH & Co. KG, Herzogenaurach, Germany), and sunflower (*Helianthus annuus* ‘AB-OR-8’) was conducted starting 2023-08-23. N = 12 Plants were harvested five times throughout the experiment (maize: day 4, 7, 10, 13, 17; sunflower: day 5, 8, 11, 14, 18; barley: day 4, 10, 13, 17, 21). To enhance efficiency of harvests, the fifteen groups were arranged block wise per harvest day, with random distribution of species within a block. As a validation, a second correlation experiment with two barley (*Hordeum vulgare*) cultivars (‘HOR 8160’ and ‘Namhaebori’) was conducted starting 2024-11-21. Twelve groups (6 harvest dates, 2 genotypes, N = 6) were formed, with block wise order based on harvest day (4, 7, 11, 14, 18 and 21 DAT), and random distribution of genotypes within a block. Prior to harvest, plant height was measured using a carpenter's square on top of the adapter as ground truth for the development of image analysis models.

The suitability of GrowScreen-Rhizo 3 to identify genotypic variation in root and shoot traits was evaluated in an experiment with barley, using 23 parental lines of a double round-robin (DRR) population of *Hordeum vulgare* [51] and the cultivar ‘Barke’ as reference line (N = 8). Starting 2022-10-13, plants grew for 24 days in a randomized complete block design, with 8 blocks with one plant per line each, *i.e.* 24 rhizotrons on three tables, resulting in a total number of 192 rhizotrons. At harvest, destructive root traits for half of the plants were manually measured. Based on image-based and manually measured trait assessment at harvest, broad-sense heritability (⁠$H_{2}$⁠) was estimated based on a method for breeding trials as described by Holland et al. and Piepho and Mӧhring [[52], [53]]. First, a random-intercept mixed model was fitted to describe each observed trait value ($y_{ij}$) for the i-th genotype in the j-th replicate as follows:

$$y_{ij}=\mu +G_{i}+\varepsilon_{ij}\text{,}$$

With μ representing the overall mean value of the trait across all observations, $G_{i}$ representing the random effect of the i-th genotype, and $\varepsilon_{ij}$ being the residual error term not variation not related to the genotype. Following this, $H_{2}$ was calculated as:

$$H^{2}=\frac{\sigma_{G}^{2}}{\sigma_{G}^{2}+\frac{\sigma_{e}^{2}}{n_{\text{rep}}}}$$

With $\sigma_{G}^{2}$ being the genetic variance of $G_{i}$, $\sigma_{e}^{2}$ being the residual variance and $n_{\text{rep}}$ being the effective number of replicates per genotype.

During the experiment, average air temperature was 24.2 ± 3.4 °C, average relative air humidity 55.0 ± 6.6 % and photosynthetic active radiation 253 ± 123 μmol m^−2^ s^−1^. Sensors on every second table revealed highest variation in microclimate between plants on the northeast versus southwest side (Fig. S6), but absolute differences between the northeast versus southwest sensors were as low as 0.8 ± 0.2 °C air temperature, 1.6 ± 0.4 % relative air humidity and 18 ± 7 μmol m^−2^ s^−1^ photosynthetic active radiation during the day.

In summary, we used N = 8 replicates for all experiments, expect of the correlation experiment in which we harvested N = 12 plants per plant species and time point in the first and N = 6 in the second correlation experiment.

### 2.10 Statistics

Data analyses were performed using R Statistical Software [54]. All presented plusminus numbers represent standard deviation. For all experiments, plants which did not germinate or cessated growth during the experiment, were considered outlier and excluded from further analyses. Statistical differences in traits between species and treatment in the movement experiment were analyzed using the built-in R function *aov* and p-values were adjusted applying a Bonferroni correction. For the calculation of $H_{2}$ in the barley genotype experiment, mixed models were derived using the *lme4* function, variance components extracted using *lmer*, *VarCorr*, *sigma*, and the *nobs* function, and for modeling utilities, *as.formula*, *model.frame*, and *nobs* were used, all from base R. Pairwise Pearson correlation coefficients (r) among traits were computed with the *cor* function of the *stats* package and visualized with the gg*corrplot* package in R. For principal component analysis, singular value decomposition was performed using the function *pcomp*, followed by a statistical comparison of genotypes using the permutation test function *adonis2*, where pseudo-*F* is modeled after the *F*-statistic from ANOVA. To allow for a meaningful comparison of pseudo-*F* values, only plants, for which root traits have been manually determined, were included in the PCA analysis.

## 3 Results

A set of experiments was performed to demonstrate the suitability of GrowScreen-Rhizo 3 for high throughput phenotyping of root and shoot traits. First, we tested monocotyledonous and dicotyledonous plant species and could demonstrate that the platform is suitable for a wide range of plant species, including crop and niche species, such as barley, wheat, maize, rapeseed, lentil, sunflower, and buckwheat, but also for wild plant species, such as evening primrose (Fig. 3). The time until roots reach the bottom of the rhizotrons depends on the plant species. While sunflower and maize reached the bottom already within two weeks, most crops typically needed three to four weeks, while slow growing plants such as evening primrose needed even up to six weeks, which of course depend on the experimental conditions.

**Fig. 4:**
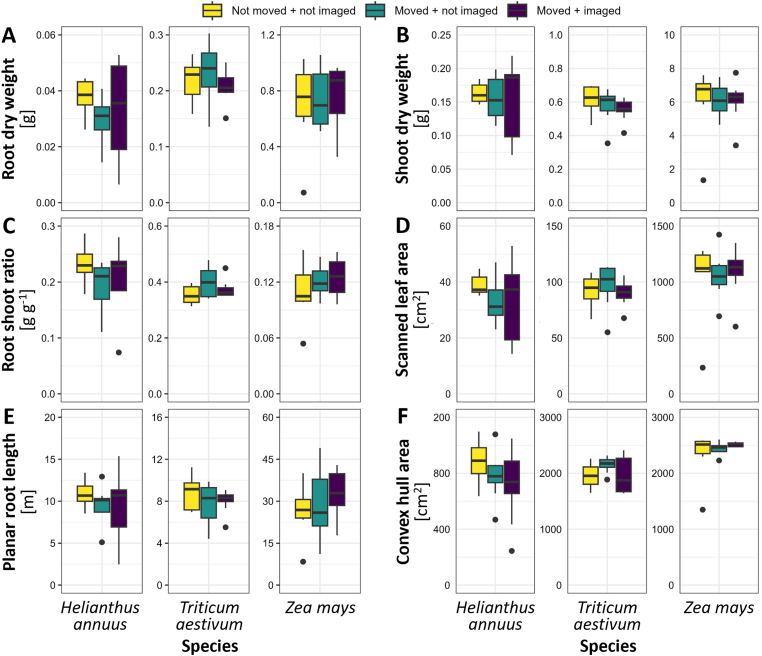
Root and shoot traits assessed at the end of the experiments (13 days after transfer (DAT) for sunflower, 25 DAT for wheat and maize) reveal no differences in plant growth when plants were moved daily and/or imaged daily. A) Root dry weight, B) Shoot dry weight, C) Root to shoot ratio based on weight, D) Scanned leaf area, E) Planar root length, F) Convex hull area of the root system of N = 6-9 plants. For all traits, a significant species effect, but no handling effect was detected (two-way-ANOVA with p < 0.05).

To validate whether the plant-to-sensor approach of GrowScreen-Rhizo 3, with daily movement and imaging, influenced root and shoot development, two experiments with plant species of different shoot sizes and geometries (wheat, maize, and sunflower) were conducted. We compared three handling protocols: 1) not moved and not imaged - rhizotrons remained in the cultivation area and were neither moved nor imaged during plant growth, 2) moved but not imaged - plants were transported to the measurement chambers daily, but were not brought inside and therefore not imaged, and 3) moved and imaged – as before, but with additional transport into the measurement chambers and imaging of roots and shoots (Fig. 4). At the end of the experiment, which was 13 days after transplanting (DAT) for sunflower and 25 DAT for wheat and maize, plants showed no significant effects of daily movement and likewise no significant effects of daily imaging (Fig. 4). Overall, we could observe a species, but no handling effects on root and shoot biomass, root to shoot ratio, leaf area, and on root traits such as root length and convex hull area (Fig. 4).

A barley genotype experiment with 192 rhizotrons was performed to assess phenotypic variation of 24 genotypes, which represent 23 parents of DRR population and the cultivar ‘Barke’. Non-invasive imaging and destructive harvest at the end of the experiment, when roots reached the bottom of the rhizotrons, were combined to quantify genotypic variation of a total of 27 root and shoot traits (Table 1). Across all measured traits, the largest variance, measured as the coefficient of variation (CV), was found for lateral root traits (CV of 53-62%). In comparison to lateral roots, the CV of seminal root traits was much lower and ranged between 18 and 30% (Table 1). The CV of root traits describing the spatial distribution of roots varied between 5% and 27% and the non-invasively measured shoot traits ranged between 31% and 41%. For the manually measured root and shoot traits at harvest, CVs between 39% and 47% were found, except for the average root diameter with a low CV of 9%. Overall, the heritability ranged between 0.52 for root system depth and 0.92 for seminal root length (Table 1).

**Table 1:** Phenotypic variation within 24 barley genotypes phenotyped using GrowScreen-Rhizo 3. Mean, standard deviation, minimum and maximum values, coefficient of variation (CV) and heritability of a total of 27 image-based and manually measured traits (N = 8) determined at the end of the experiment.

Method	Trait	Unit	Average	±SD	Minimum	Maximum	CV (%)	Heritability
***Daily automatic imaging of plants***								
Shoot image analysis	Predicted leaf area (LA_predicted_)	cm^2^	149.63	61.17	18.01	340.93	40.88	0.85
	Plant height	cm	28.42	8.92	5.12	58.32	31.40	0.89
	*Shoot growth rate*	*cm*^*2*^*d*^*−*^*^1^*	6.50	2.66	0.78	14.82	40.90	0.85
Root image analysis	Root system depth	cm	73.07	3.96	39.41	74.75	5.45	0.52
	Root system width	cm	28.21	5.98	9.62	36.09	21.72	0.62
	Root system convex hull area	cm^2^	1612.46	421.59	338.67	2427.01	27.06	0.61
	Planar root length	cm	767.49	243.81	149.41	1474.65	33.15	0.67
	*Root system growth rate*	*cm d*^*−*^*^1^*	32.68	9.53	11.66	63.15	28.96	0.67
	Seminal root length	cm	505.88	150.79	123.82	940.10	29.81	0.92
	*Proportion of seminal roots*	*cm cm*^*−*^*^1^*	0.68	0.12	0.26	0.92	17.83	0.81
	*Seminal root growth rate*	*cm d*^*−*^*^1^*	21.31	6.44	5.38	40.04	30.24	0.92
	Lateral root length	cm	261.61	139.07	25.33	775.46	53.16	0.73
	*Proportion of lateral roots*	*cm cm*^*−*^*^1^*	0.53	0.33	0.08	2.79	61.92	0.80
	*Lateral root growth rate*	*cm d*^*−*^*^1^*	23.27	12.24	2.40	58.37	52.62	0.70
	*Lateral to seminal root length ratio*	*cm cm*^*−*^*^1^*	0.32	0.12	0.08	0.74	37.19	0.81
Combined image analysis	*Image-based root to shoot ratio*	*cm cm*^*−*^*^2^*	5.65	2.08	3.20	16.97	36.74	0.81
***Manual measurements at harvest***								
Weighing	Shoot fresh weight	g	5.54	2.62	0.44	16.11	47.30	0.86
	Shoot dry weight	g	0.71	0.29	0.09	1.48	41.15	0.85
	Root dry weight	g	0.26	0.11	0.05	0.68	43.65	0.79
	*Plant biomass*	*g*	0.97	0.38	0.15	1.87	39.12	0.83
	*Root to shoot ratio*	*g g*^*−*^*^1^*	0.38	0.14	0.16	1.30	36.54	0.78
Manual assessment	Length of the longest leaf	mm	467.89	61.04	242.00	646.00	13.05	0.79
	Tiller number		3.23	1.74	0	8	53.728	0.87
Leaf area meter	Scanned leaf area	cm^2^	161.02	71.69	17.24	436.18	44.52	0.86
Root scanning	Scanned root length	cm	3102.77	1304.65	647.76	7016.18	42.05	0.61
	Average root diameter	mm	0.58	0.05	0.50	0.76	8.58	0.87
	*Scanned root to shoot ratio*	*cm cm*^*−*^*^2^*	5.35	2.14	2.78	19.69	40.06	0.84

**Fig. 5:**
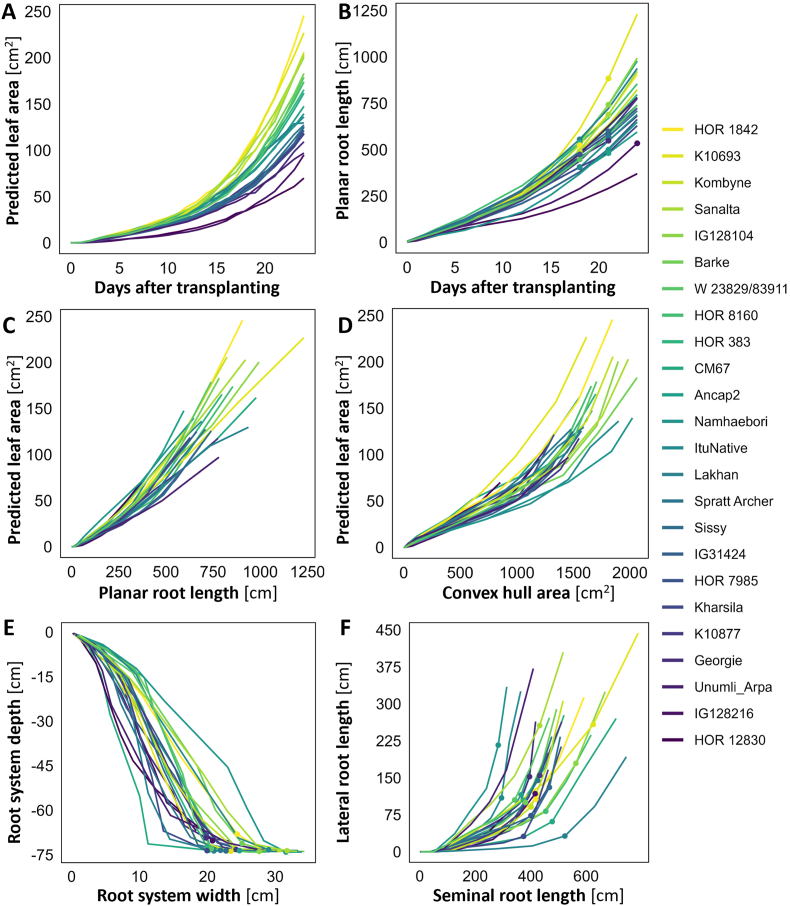
Phenotyping of root and shoot growth of 24 barley genotypes over time. A) Predicted leaf area, B) Planar root length, C) Predicted leaf area in relation to planar root length, D) Predicted leaf area in relation to convex hull area of the root system, E) Changes in root system depth in relation to root system width, F) Growth of lateral roots in relation to seminal root length. Mean values of each genotype are plotted (N = 8). Dots indicate the measurement time point when roots of more than four replicates per genotype reached the bottom of the rhizotrons.

**Fig. 6:**
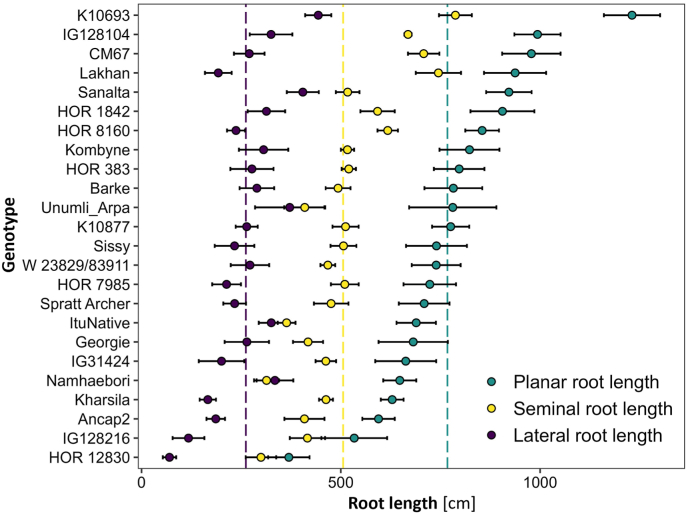
Genotypic variation in planar, seminal, and lateral root length of 24 barley genotypes 24 days after transplanting. Dots represent averages ±SE of N = 8 replicates per genotypes. Dashed lines indicate average values per root types among all genotypes.

Daily imaging allowed to monitor genotypic variation in plant development over time (Fig. 5A and B). Predicted leaf area showed exponential growth in all genotypes and varied between 60 and 240 cm^2^ at the end of the experiment. Planar root length determined from the images increased likewise in all genotypes to maximum values between 350 and 1250 cm (Fig. 5B). Although genotypes generally showed steady shoot and root growth, the temporal analysis also revealed that some genotypes displayed changing growth rates, *i.e.* above-average predicted leaf area or planar root length after initially slow growth rates, or vice versa. The simultaneous image-based assessment of predicted leaf area and planar root length revealed genotypic variation in the relation of aboveground to belowground growth (Fig. 5C), which was even further emphasized when comparing predicted leaf area to convex hull area of the root system (Fig. 5D). We found genotypes with a similar leaf area size, but almost two times differences in root system size (Fig. 5C and D). Likewise, the spatial analysis of root growth revealed genotypic variation in the use of substrate area (Fig. 5E and F). Genotypes varied in terms of the ratio between root system width and depth, which is indicated by a much earlier horizontal growth in some genotypes, resulting in root systems that were two-to threefold wider (Fig. 5E). The asymptotic behavior towards the end of the experiment occurs, when roots reach the bottom of the rhizotrons. Frequent imaging of root systems also allowed to characterize the developmental transition from seminal to lateral root formation (Fig. 5F). While some genotypes show very early formation of lateral roots, others showed this rather late. At the end of the experiment, genotypes vary in planar root length, with root length up to threefold higher in fast-growing compared to slow-growing genotypes (Fig. 6). Although genotypes showed generally higher seminal than lateral root length (exception: genotype Namhaebori), there is considerable genotypic variation between investment in seminal or lateral root length (Fig. 6).

**Fig. 7:**
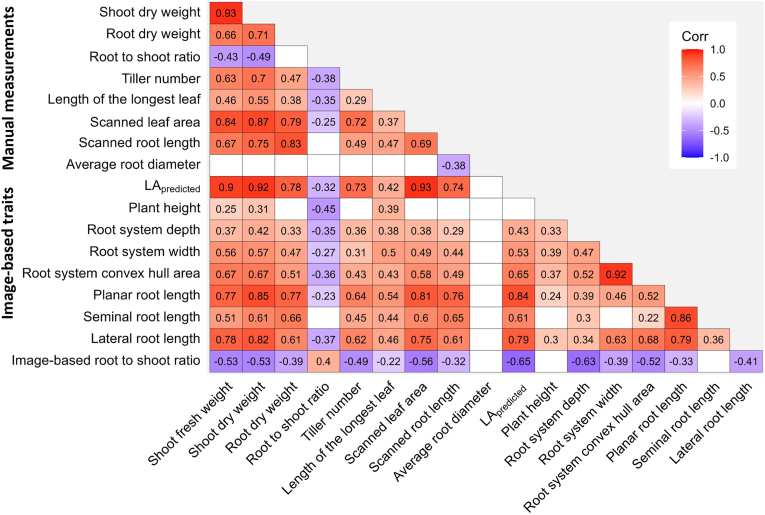
Correlation analysis for manually measured and image-based shoot and root traits gathered in an experiment with 24 barley genotypes. Values represent coefficient of determination (R^2^). White boxes indicate non-significant correlation.

A correlation analysis of all traits assessed at the end of the experiment with 24 barley genotypes revealed high correlations between manually measured and image-based traits (Fig. 7). It should be noted that the correlation between predicted leaf area and scanned leaf area may be overestimated, as the dataset was used for training the image analysis model. However, similarly high correlations of r^2^ = 0.95 between predicted and scanned leaf area were also observed in further barley experiments, and predicted leaf area also showed an exceptionally high correlation to the independently assessed shoot dry weight (r^2^ = 0.92). The manually measured length of the longest leaf, which is often used for non-destructive measurement of the growth of monocotyledonous plants, is a much weaker indicator of plant growth, with r^2^ = 0.55 for shoot dry weight and r^2^ = 0.37 for scanned leaf area. Planar root length correlated very well with destructively scanned root length (r^2^ = 0.76) as well as with the root dry weight (r^2^ = 0.77, Fig. 7, S7). Furthermore, the planar root length exhibited high correlation to shoot traits, such as shoot fresh and dry weight, and predicted leaf area (r^2^ = 0.77, 0.85 and 0.84, respectively) and to root traits based on length measurements, such as seminal and lateral root length (r^2^ = 0.86 and 0.79, respectively, Fig. 7). In contrast, the correlation between planar root length and plant height is low (r^2^ = 0.24) and low to moderate to spatial root system traits, such as root system depth and width and convex hull area (r^2^ = 0.39, 0.46 and 0.52, respectively, Fig. 7). The correlation is low between different root classes e.g. r^2^ = 0.36 between seminal and lateral root length (Fig. 7). Interestingly, average root diameter showed no significant correlations to other traits, except for a moderate, negative correlation with scanned root length (r^2^ = −0.38).

**Fig. 8:**
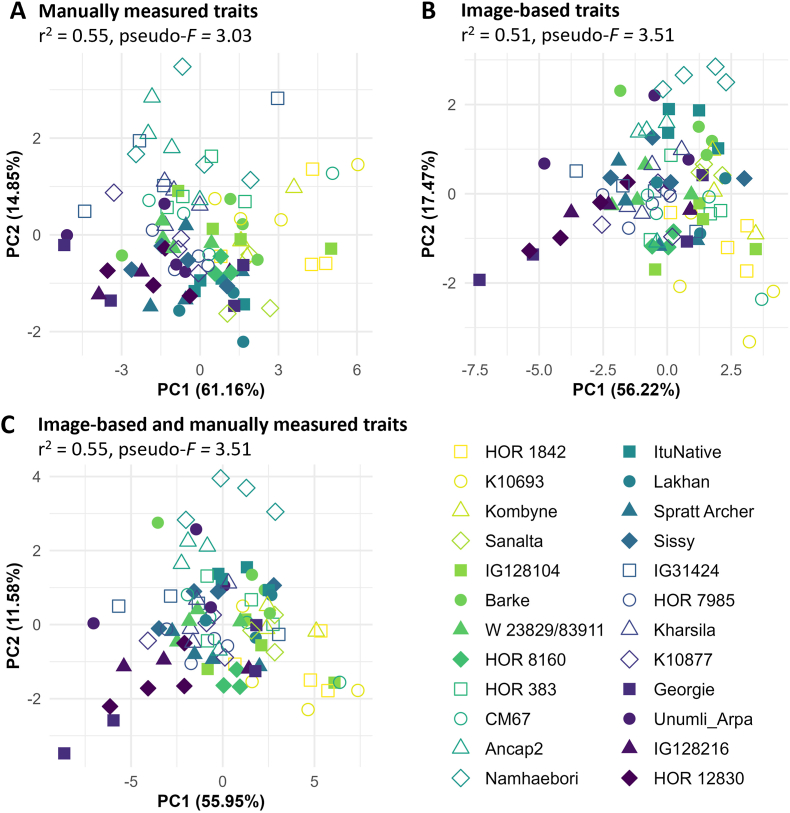
Principal Component Analysis (PCA) of traits measured in GrowScreen-Rhizo 3 in an experiment with 24 barley genotypes. Genotypic variation is demonstrated in A) manually measured traits, B) image-based traits and C) combined manually measured and image-based traits based on the first two principal components. Significant separation between genotypes (N = 4 plants per genotype) is indicated by pseudo-*F* based on permANOVA.

To further demonstrate the suitability of manually measured and image-based traits for deciphering genotypic variation, principal component analysis (PCA) was performed, which included either manually measured, image-based, or combined manually measured and image-based traits (Fig. 8). For all three variants, a high percentage of the total variance was explained by the first two principal components, namely 76%, 74%, and 68% for manually measured, image-based and combined traits, respectively. For manually measured traits, the most relevant loadings for PC1 (61%) are shoot fresh and dry weight and scanned leaf area, and for PC2 (15%) average root diameter, scanned root length, and length of the longest leaf. For image-based traits, the most relevant loadings for PC1 (56%) are planar root length, predicted leaf area, and lateral root length, and for PC2 (17%) seminal root length, root system width, and shoot height. When considering combined manually measured and image-based traits, highest drivers for the observed variation were shoot dry weight, predicted leaf area, and planar root length for PC1 (56 %), and shoot height, root system width, and average root diameter for PC2 (12%), respectively. Many genotypes showed distinct clustering, especially when incorporating image-based traits, e.g. close clustering of the plants of the genotypes Kharsila, HOR 12830, Naemhaebori, and others. This is also confirmed by comparing the PCA scores of the first two principal components by permANOVA analysis, which confirmed a significant clustering of genotypes with pseudo-*F* values of 3.03 for manually measured traits, but higher pseudo-*F* values of 3.51 both for exclusively image-based traits and combined manually measured and image-based traits. Including manually measured traits in the analysis did not contribute to better distinguish genotypic differences.

## 4 Discussion

The last two decades have seen an increased interest in root research to unravel basic root developmental programs and regulatory gene networks orchestrating physiological and plastic responses to growth-limiting conditions [4,55]. Notably, trait-based root research has led to the definition of possible phenotypic ideotypes for specific environmental and edaphic conditions potentially relevant to plant breeding programs [[56], [57], [58], [59]]. In this respect there is significant interest in contributing improved methods to enable the screening of diverse genetic resources and characterize roots phenotypic variation and plasticity to the environment. Several soil-based root phenotyping platforms using clear pots or rhizotrons have been developed to study root traits under close to natural soil conditions (Table S2). Root traits such as root length, types, and angles describe global root morphology and have been shown to exhibit moderately high heritability in both field and controlled environment. The various phenotyping platforms accommodate experiments with different scopes ranging from the characterization and screening of a few target traits such as variation in seminal root number and angles in contrasting wheat accessions [32] or to dissect the genetic control of nodal root angles in sorghum [34], to variation of 47 root traits in wheat core association mapping panels ([39]; Table S2). High throughput root phenotyping platforms enabled GWAS studies, e.g. for root angles and rooting depth in an *Arabidopsis* diversity panel consisting of 96 genotypes [40]. Experimental protocols using these platforms have been validated to reveal genetic and phenotypic variation in root development e.g. genetic and phenotypic variation in upland cotton varieties [35] and drought stress responses of maize accessions [36].

To date, to the best of our knowledge, only a few platforms have been developed that combine root and shoot phenotyping. The first platform that enabled simultaneous acquisition of shoot and root images over time was GrowScreen-Rhizo 1, which was first used for combined analysis of root and shoot traits of six species and addressing the effect of soil compaction on root growth of barley and maize [31]. In a sensor-to-plant approach, it featured 72 large rhizotrons and was key to study the integrated and dynamic responses of root and shoot traits of cereals and legume species to reduced water availability [[60], [61], [62], [63]], nitrogen limitation [64], as well genotypic variation in shoot and root traits in wheat [65,66]. It is worth noting that the studies mentioned above also included manual measurements, e.g. number of leaves and leaf elongation rates, physiological measurements, e.g. gas exchange [60] and destructive measurements at harvest. However, the throughput of GrowScreen-Rhizo 1 was limited by space and time requirements, the excessive rhizotron weight required a high number of personnel for experimental setup, and the platform design did not allow for modular expansion of the setup. Subsequently, platforms that by default integrated root and shoot imaging for the same individual plants were presented by Jeudy et al. ([24], rhizotubes with semi-permeable membrane to force visibility of the entire root system), Zhang et al. ([37], rhizotrons), and Shi et al. ([33], ‘Rhizo-pots’, large pots with tilted transparent side). After GrowScreen-Rhizo 1, we designed a second root phenotyping prototype featuring a plant-to-sensor approach. The sensor-to-plant concept of GrowScreen-Rhizo 1 with fixed plant positions and a moveable measurement chamber limited the flexibility and capacity of the platform. Consequently, a new plant-to-sensor concept was tested in the next prototype system, namely GrowScreen-Rhizo 2 [41]. In GrowScreen-Rhizo 2, rhizotrons were transported in magazines along conveyor belts to a measurement chamber with a fixed position. In addition to the different transport system the size of rhizotrons was reduced from 90 × 70 × 5 cm as used in GrowScreen-Rhizo 1 to 80 × 40 × 5 cm in GrowScreen-Rhizo 2 to facilitate the handling of the rhizotrons, which also increased the resolution of the root images from 230 to 142 μm per pixel (Table S2, [[31], [41]]). The concept of GrowScreen-Rhizo 2 and was validated in an experiment with localized digestate fertilization of *Sida hermaphrodita* plants [41]. Based on the experiences of both prototype systems with capacities of 72 and 80 rhizotrons, respectively, we designed the high throughput phenotyping platform GrowScreen-Rhizo 3 with a capacity of 896 rhizotrons. GrowScreen-Rhizo 3 prioritizes both capacity and throughput and enables highly standardized high throughput whole plant phenotyping experiments fulfilling future experimental needs. First, we maximized capacity by building a plant cultivation area comprising all 896 rhizotrons/plants. To our knowledge, this capacity is so far larger than any other rhizotron-based phenotyping platform in the public domain. The capacity of most soil-based root phenotyping platforms ranges from 50 to 200 plants (Table S2). Only one platform using rhizotrons features a capacity of 500 plants, but it requires manual imaging of roots [34]. In our experience, a capacity of a few hundred plants is necessary to accommodate large experiments as required by association mapping studies including possible experimental treatments or conducting multiple and different experiments at the same time. Second, we addressed the need of increasing throughput by construction of four identical measurement chambers, instead of one chamber as in both prototype systems, which allows whole-plant imaging of all 896 rhizotrons in approx. 6 h. Third, we adapted the rhizotrons approved in GrowScreen-Rhizo 2 to improve stability while reducing weight. The rhizotron size of 80 × 40 × 5 cm enabled to further improve root image resolution to 116 μm per pixel and is at the upper range of rhizotron sizes of published phenotyping platforms, which vary between 20 and 110 cm in height (Table S2). Fourth, we followed the plant-to-sensor approach of our second prototype with moving rhizotrons in magazines through the platform, but instead of conveyor belts we used AGVs for transport. AGVs are successfully used in many industrial processes for automated transport of goods without human intervention, for example in warehouses and manufacturing facilities. In plant phenotyping, AGVs are still rarely used (e.g. [39]) despite offering advantages compared to conveyor belt systems. While a conveyor belt is fixed to the ground and plants must be lined up one behind the other and always moved along the same paths, the paths of AGVs can be adapted easly to match specific experimental requirements. AGVs furthermore grant flexible access to plants and allow to randomize plants over time. Multiple AGVs can operate in parallel and perform different tasks simultaneously, for example delivering plants to parallel working measurement chambers for imaging for increasing the throughout. In general, the modular design of the platform GrowScreen-Rhizo 3 can be tailored to specific requirements and constraints, while enabling flexible and efficient utilization of available space. The modular approach allows to built small scale systems with one measurement station, as well as to scale up numbers of AGVs and measurements stations to built large platforms with exceptionally high throughout, as presented with GrowScreen-Rhizo 3. Fifth, we developed a novel automated weighing and watering system that irrigates each rhizotron individually with a pre-defined amount or to a pre-defined target weight. This system reduces variability potentially introduced by manual irrigation protocols and enables exposing plants to reduced water availability as intended in simulated drought experiments. Finally, we paid great attention to develop a semi-automated filling line for assembly of rhizotrons. The combination of a tray filler, load cells, and a specialized press reduced variation in substrate density to for example 1.8% across rhizotrons of the barley genotype experiment. We are not aware of any other root phenotyping platform that enables such standardized filling of growth containers with comparable efficiency.

Overall, in this work, we presented data demonstrating the suitability of GrowScreen-Rhizo 3 for high throughput phenotyping of root and shoot traits. A range of experiments using different plant species has been completed using GrowScreen-Rhizo 3. All investigated species have established well in rhizotrons and showed shoot morphology comparable to pot-grown plants (Fig. 3). Root system architecture is ideally analyzed before roots reach bottom or sides of the rhizotrons, as this may affect root elongation [67]. Experimental duration is determined by when the first roots axis reach the bottom of the rhizotrons. The exact duration is dependent on plant species and environmental conditions, ranging from nine days for fast-growing sunflower hybrids to several weeks for slow-growing plants such as evening primrose. For species with prostrate growth habit, use of shoot retainers is recommended to avoid leaf damages during automated handling of rhizotrons at the measurement chambers.

Plants as sessile organisms do not experience alteration in position. We conducted experiments designed to evaluate the potential effect of moving plants for imaging and watering routines. These experiments with maize, wheat, and sunflower showed that movement of plants by AGVs and daily imaging did not significantly affect plant growth (Fig. 4). The moderate acceleration and deceleration employed by AGVs and conveyor systems in GrowScreen-Rhizo 3 did neither affect aboveground nor belowground plant development when compared to plants that were not moved at all or moved by AGVs but not taken out of the magazines (Fig. 4). Likewise, no effects of plant movement on root traits and leaf area ([22], GrowScreen-Agar) or aboveground biomass and metabolite profiles ([42], IPK LemnaTec Scanalyzer system) of *Arabidopsis* were observed in platforms following similar plant-to-sensor measurement routines. Furthermore, we did not observe any effect of the imaging protocol itself, *i.e.* the short exposure of root systems to light (Fig. 4). Light has been shown to affect root growth of *in vitro* grown plants [68,69], and even short light flashes have been shown to trigger ROS formation in roots of *Arabidopsis* [70]. Therefore, the design of GrowScreen-Rhizo 3 magazines prevents illumination of rhizotrons at any time during plant cultivation. Exposure to light during imaging of rhizotrons is minimized to a few seconds and did not result in any phenotypic effect on root and shoot traits (Fig. 4).

We demonstrated that GrowScreen-Rhizo 3 can be routinely used to study diverse genotype panels. Genotypic variation in 27 root and shoot traits assessed in our phenotyping experiments was revealed for 24 studied barley genotypes (Table 1). This genetically and phenotypically diverse population of barley inbred lines has previously been shown to vary in shoot phenology [71] and photosynthesis-related traits [72]. We observed a notably high heritability of traits, with maximum values for seminal root length (Table 1). Seminal root traits have been shown to have high heritability in early breeding experiments [73] as well as recent phenotyping experiments [[74], [75], [76]]. However, rhizotrons used in these studies were smaller, limited the experimental duration, and did not allow to observe the formation of lateral roots.

A major advantage of the high degree of automation of GrowScreen-Rhizo 3 is the possibility to image plants with high temporal resolution. Daily imaging of plants allowed to distinguish differences in growth patterns between genotypes. The high resolution observation of predicted leaf area and planar root length revealed variation in the early establishment and consecutive growth rates. Some genotypes showed initially rather slow establishment followed by fast growth, while others established well, followed by slow growth. Consequently, growth in the first days was not necessarily indicative for plant size at the end of the experiment (Fig. 5A and B). Differences in the early establishment between genotypes may be related to genotypic variation in seed size or resources, which are known to affect plant performance during first days after germination [77]. Combining simultaneous measurements of shoot and root growth showed genotypic variation in biomass allocation between above- and belowground (Fig. 5C and D). Temporal analyses also revealed genotypic variation in the exploitation of soil space, with narrower root systems (Fig. 5C) indicating lower root angles, a trait known to strongly increase tolerance to abiotic stresses such as drought [76] or soil acidity [74]. Likewise, genotypes strongly vary in the developmental transition from seminal to lateral root formation (Fig. 5D), which is under genetic control and modified by environmental factors. At the end of the experiment, planar root length and distribution of root types distinctly vary between genotypes (Fig. 6), indicating the advantage of the large dimensions of GrowScreen-Rhizo 3 rhizotrons to reveal lateral root traits which could not be derived in earlier experiments with smaller rhizotrons or clear pots [74,76].

GrowScreen-Rhizo 3 features combined root and shoot image analysis and additionally enable manual measurements during or at the end of the experimental period. However, non-automated measurements are laborious and limit the throughput [42,78]. It is therefore advantageous to reduce the number of manual measurements, given established automated measurement procedures. The combination of several shoot cameras with novel algorithms to derive predicted leaf area enables a reliable representation of leaf area (Fig. 7), which enables to non-destructively monitor shoot growth with daily resolution. Likewise, high correlations between image-based planar root length and laboriously, manually determined scanned root length emphasize its suitability to characterize root traits non-invasively (Fig. 7, S7). Furthermore, image-based analysis of root types provides inside into root growth dynamics that cannot be achieved by destructive harvests. In that regard, the high variability in proportion of seminal root length between genotypes contributes to a low relationship between seminal and lateral root length (Fig. 7).

Finally, we conducted a comparative principal component analysis (PCA) to assess the suitability of manually measured destructive traits and image-based, non-invasively assessed traits to distinguish barley genotypes (Fig. 8). Distinct clustering of genotypes such as Kharsila, HOR 12830, and Naemhaebori indicates the general suitability of GrowScreen-Rhizo 3 to reveal genotypic variation, and most relevant loadings reveal which traits explain most of the observed variation. Interestingly, despite the observed high variation in image-based root traits, manually measured root traits play a minor importance to distinguish genotypes when considering manually measured traits only. When considering image-based traits only, root traits were of higher relevance than predicted leaf area, which is in line with earlier studies on genotypic variability in root traits [[74], [75], [76]]. Combining both manually measured and image-based traits did not further improve clustering of genotypes, indicating that acquiring manually measured traits hardly adds relevant information to represent the genotypic diversity of the studied barley population. These results indicate that in future experiments, the assessment of manually measured traits can be reduced in certain experiments without losing information. One exception is shoot dry weight, which had highest relevance when considering both manually measured and image-based traits, but is also the least laborious manually measured trait and can be easily determined even in large experiments with hundreds of plants. Reducing the traits to be assessed is in line with recommendations by Poorter et al. [78] to focus on a selection of meaningful traits. In our case, this will contribute to expand experiment size and maximum occupancy of GrowScreen-Rhizo 3.

Based on our experience, we envision future root phenotyping platforms to encompass possibilities to specifically address root-soil interactions and rhizosphere level processes (e.g., microbiome level and root exudation analyses). So far, to the best or our knowledge, there is only one high-throughput platform specifically designed to study root-microbe interactions [24]. Technological advances must be made to be able to study root microbe interactions with high spatial and temporal resolution of measurements and enable complementing sampling with high throughput. Regardless of the focus, we stress that increased capacities and throughput of phenotyping facilities bring about the necessity to streamline and optimize quantification of traits, to standardize quality control assessment and to advance statistical analyses. In this respect, the routine use of dedicated machine learning methods and AI models will become essential for image analysis and data exploration. Finally, we consider strengthening the link between indoor phenotyping approaches to field trials essential to relate root traits measured at relatively early growth stages to biomass productivity and crop yield.

## 5 Conclusion

GrowScreen-Rhizo 3 presented in this article is a unique platform for simultaneous high throughput phenotyping of shoots and roots. We demonstrated the utility of our protocols across a wide variety of plant species, including mono- and dicotyledonous crop, niche, and wild plant species. The platform allows to phenotype whole plant populations non-invasively e.g. for GWAS analysis and to assist breeding pipelines. In combination with the application of different abiotic and biotic stresses, the platform will allow for example to identify candidate genotypes with altered stress tolerance, with improved plant productivity or resource use efficiency (water or nutrients). Access to GrowScreen-Rhizo 3 platform is available via national and international plant phenotyping networks such as the German Plant Phenotyping Network (DPPN).

## Authors contribution

HL, AP, and KAN have made substantial contributions to the conception and design of the phenotyping platform GrowScreen-Rhizo 3 and of the rhizotrons. HL, AP, and OM designed the rhizotron filling line. HL, AP, and SA contributed to the construction of the platform. HL, AP, and JW developed the software to run the phenotyping system. JW, CE, and MML developed the image analysis pipeline. LJF, HL, AG, and KAN developed experimental procedures and protocols. LJF, HL, SJ, AG, JL, FF, MML, and KAN designed and performed the experiments and LJF, SJ, and FF analyzed the phenotypic data. LJF, FF, and KAN worked on interpretation of data. LJF, SJ, CE, FF, and KAN drafted the manuscript. All authors read and approved the final manuscript.

## Data

The data including the metadata are published at Jülich DATA (https://doi.org/10.26165/JUELICH-DATA/BPSWGG and https://doi.org/10.26165/JUELICH-DATA/SOJ6SQ).

## Funding

The development of the GrowScreen-Rhizo 3 device was supported by the German 10.13039/501100002347Federal Ministry of Education and Research (German Plant Phenotyping Network (DPPN), 10.13039/501100002347BMBF Fkz. 031A053). Furthermore, the authors acknowledge the support of Forschungszentrum Jülich GmbH in the 10.13039/501100009318Helmholtz Association. The experiments were conducted as part of third-party projects funded by the 10.13039/501100000780European Union Grant (CROPINNO, grant agreement No. 01059784) and by the German 10.13039/501100002347Federal Ministry of Education and Research (DPPN-Access, Fkz. 031B121; MAZE3, Fkz. 031B1301E; InnoWert Fkz. 03WIR3024B). SJ was funded by CSC scholarship (grant No. 202107720055).

## Declaration of competing interest

The authors declare that they have no known competing financial interests or personal relationships that could have appeared to influence the work reported in this paper.
