# Supplementary material for: GrowScreen-Rhizo 3 - automated large-scale high throughput greenhouse phenotyping of plant root and shoot development
Source: Plant Phenomics. 2026 Apr 23;8(2):100213. doi: 10.1016/j.plaphe.2026.100213 (PMC13158764; doi:10.1016/j.plaphe.2026.100213)
Supplement: Multimedia component 1 [file mmc1.pdf]

Supplementary information

GrowScreen-Rhizo 3 - automated large-scale high throughput greenhouse phenotyping of plant root and shoot development

Laura Verena Junker-Frohn, Henning Lenz, Shiyan Jia, Alexander Putz, Jens Wilhelm, Constantin Eiteneuer, Sascha Adels, Olaf Mück, Anna Galinski, Jonas Lentz, Fabio Fiorani, Mark Müller-Linow, Kerstin A. Nagel

The following Supporting Information is available for this article:

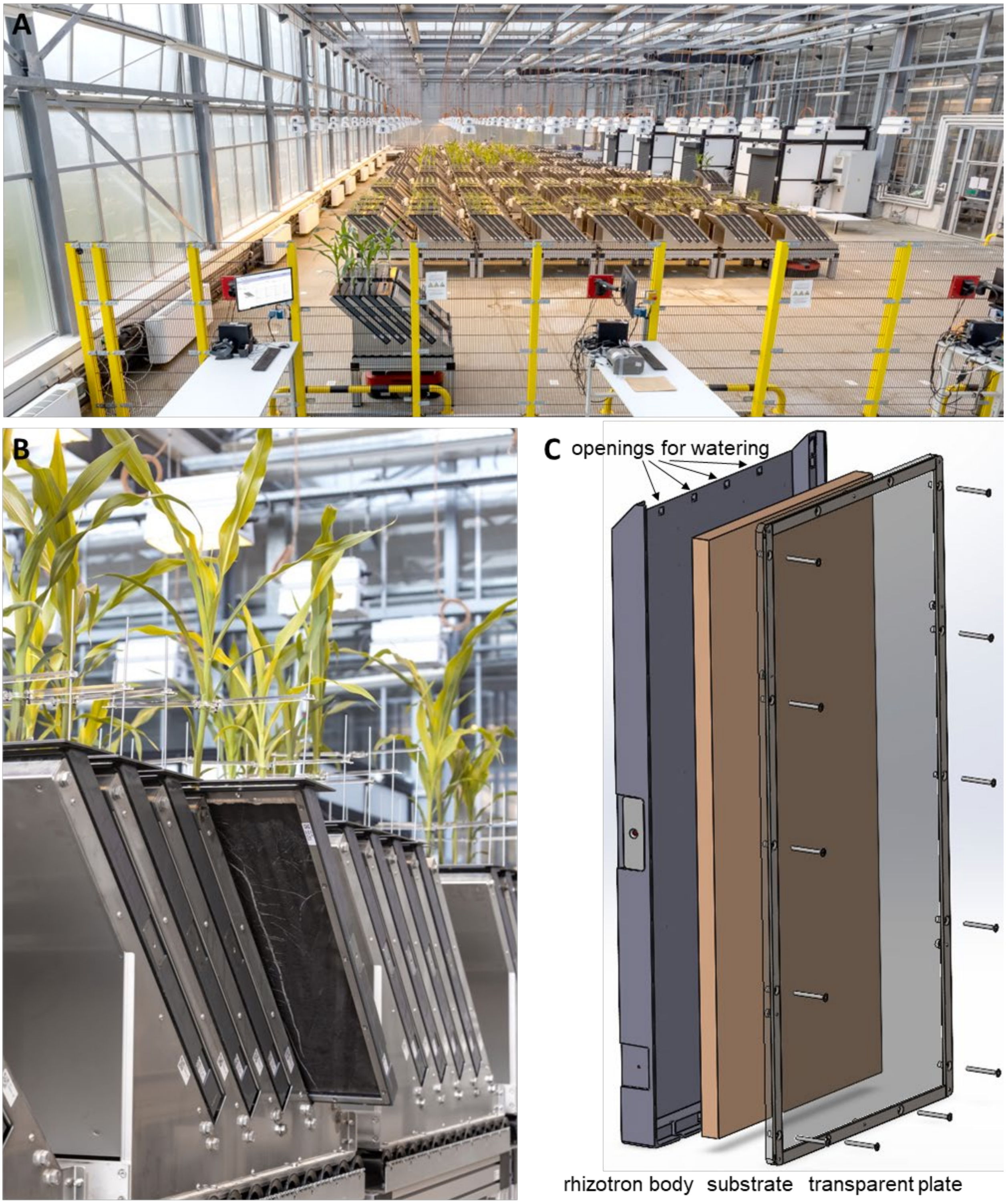

Fig. S1: GrowScreen-Rhizo 3 platform. A) Complete view of the greenhouse compartment with plant cultivation area at the center, four measurement chambers on the right side, three presentation stations in front of the fenced area, with one table in position for manual shoot measurements. B) Side view of tables with one rhizotron pulled outside to display the root system. C) Schematic drawing of rhizotrons: lightweight, durable plastic rhizotron bodies, evenly compressed substrate and transparent polycarbonate plate, which is fixed with twelve screws.

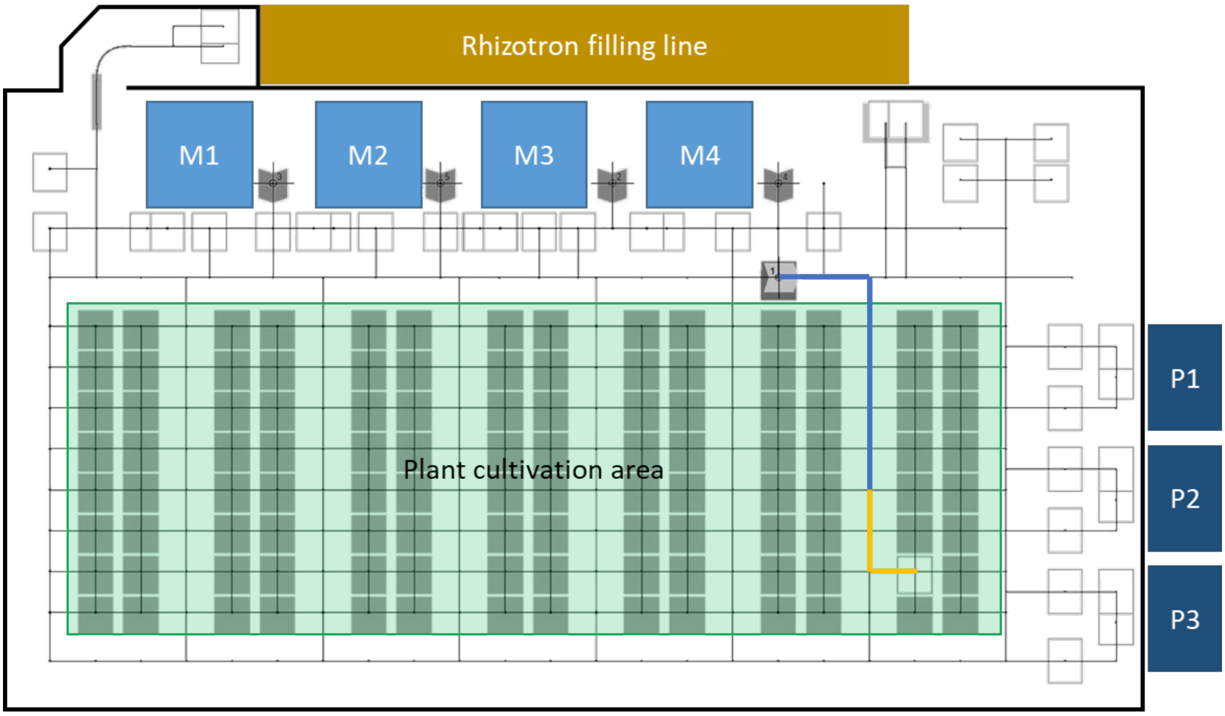

Fig. S2: Schematic drawing of the GrowScreen-Rhizo 3 platform with the plant cultivation area (marked in green), 4 measurement chambers (M1-4, marked in light blue), 3 presentation stations for sowing, manual measurements, and shoot harvests (P1-3, marked in in dark blue) and the rhizotron filling line (marked in brown). 5 AGVs (marked in grey) transport tables with rhizotrons within the platform. As one example, AGV 1 is transporting a table to a storage place in the plant cultivation area. The path of the AGV 1 shown in blue represents that this path is reserved for AGV 1 (no other AGV is allowed to use this path while it is reserved), while the yellow path is planned but not yet reserved for this AGV.

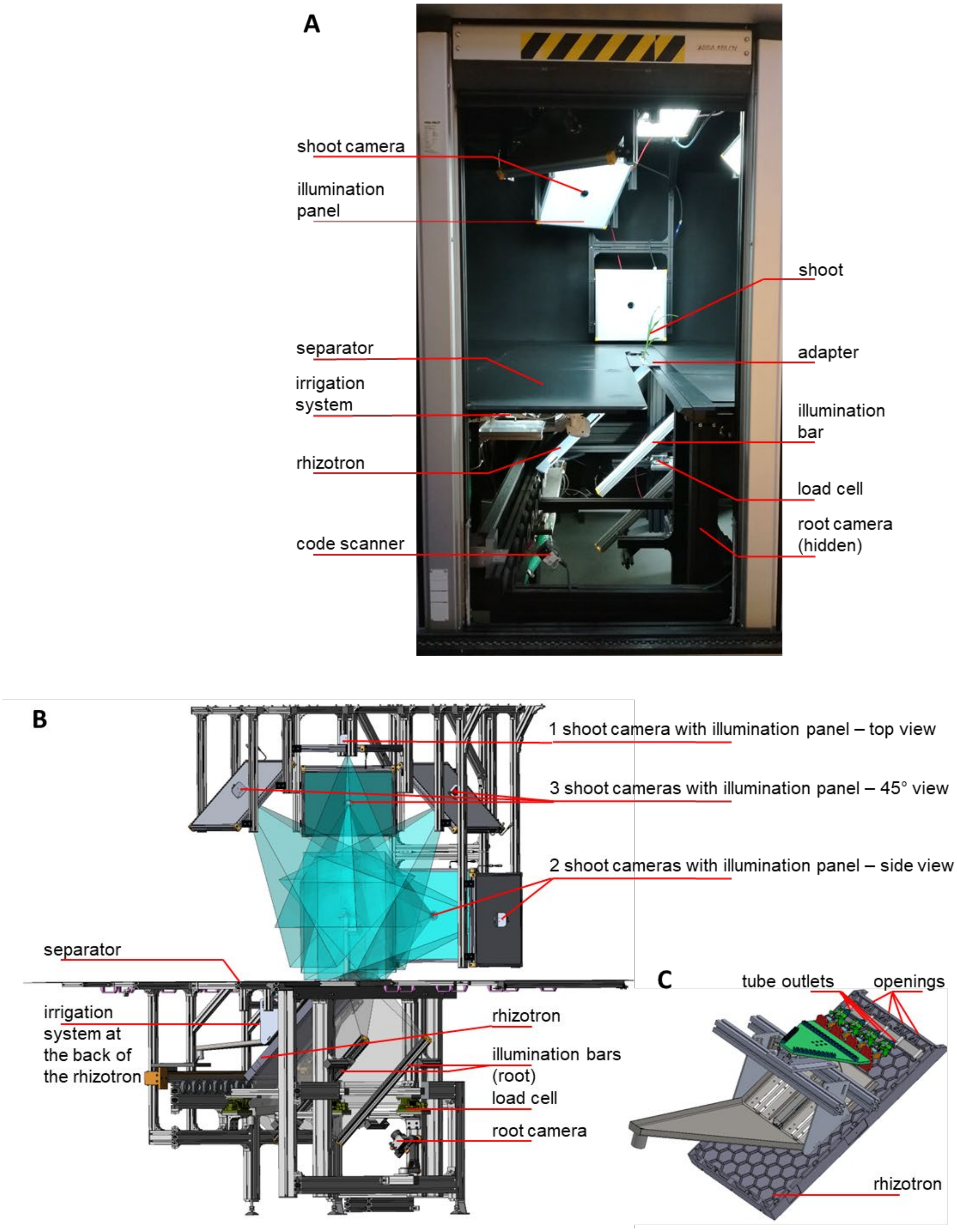

Fig. S3: Details of GrowScreen-Rhizo 3 measurement chamber. A) Image of measurement chamber interior space with root and shoot cameras, illumination bars and load cells. B) Schematic drawing of the measurement chamber with positions of cameras and illumination panels/ bars, load cells and irrigation system. C) Schematic drawing of the irrigation system (pump and hoses left out for clarity).

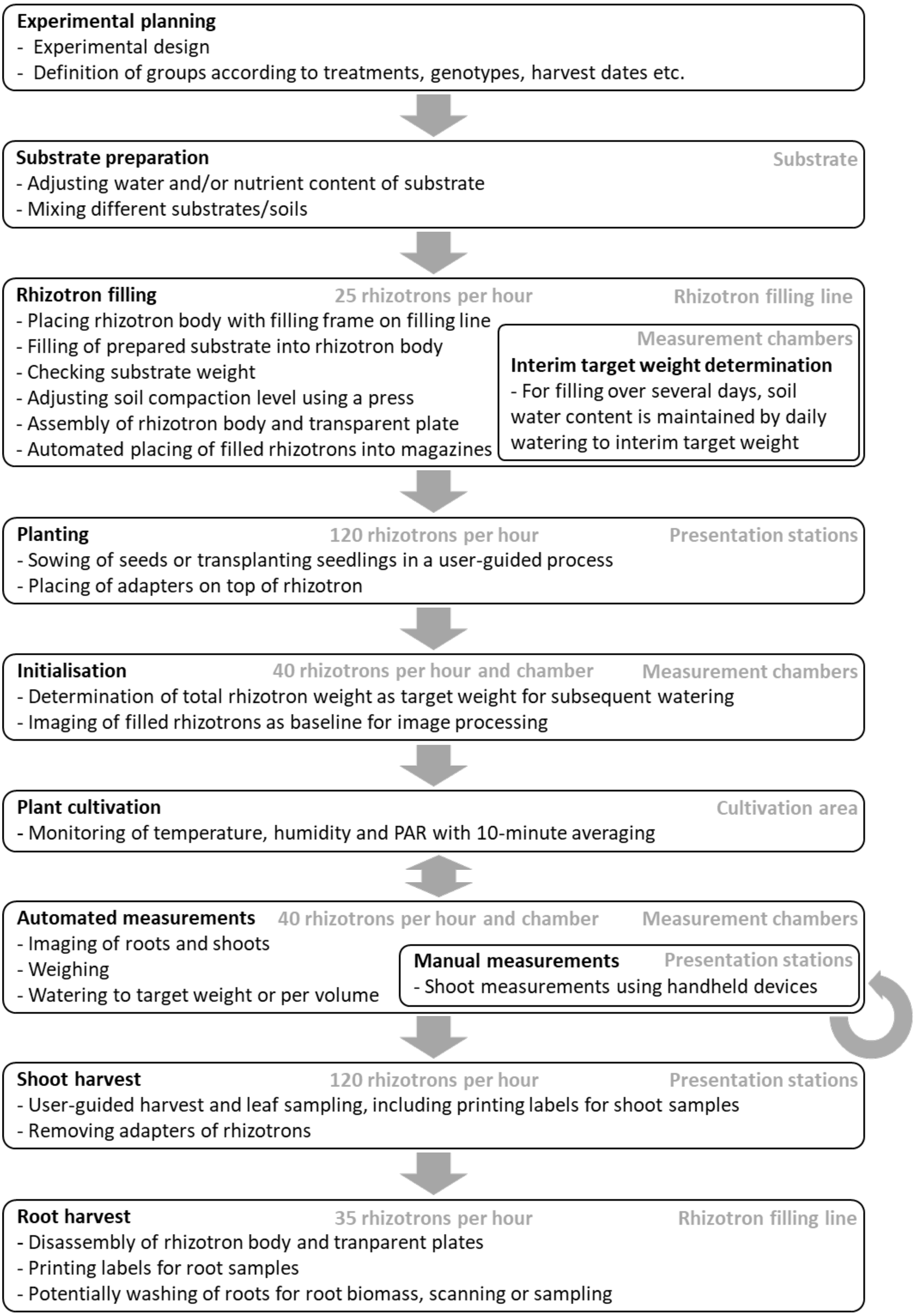

Fig. S4: Flowchart of the experimental procedures of GrowScreen-Rhizo 3 phenotyping experiments. For each step of the experiment, the maximum throughput is given. Where human interaction is necessary, 2-3 people are involved.

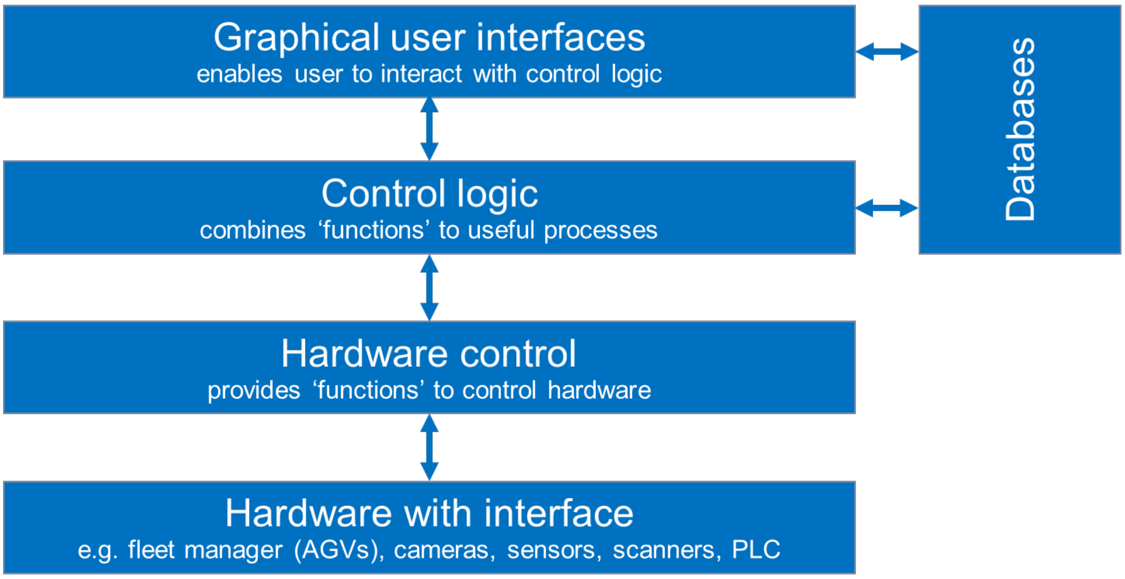

Fig. S5: Hard- and software layers to control GrowScreen-Rhizo 3. The hardware layer contains for example the fleet manager to control the AGVs, cameras for root and shoot imaging, sensors recognizing positioning, and scanners reading identification labels at various stations etc., and the programmable logic controllers (PLC) interfacing further automated hardware. The 'hardware control' layer wraps the provided hardware functionality into simple actions (e.g. pulling a rhizotron into a measurement chamber, weighing a rhizotron, triggering the imaging and watering). The 'control logic' layer combines these actions as required for a specific process (e.g. weighing a rhizotron first followed by imaging with root and shoot cameras and weight-dependent watering). The 'control logic' is linked to the graphical user interface, which displays information about the current status and guides the users through all manual processes. Both control logic and user interface are linked to databases, in which all identification, location, and experimental workflow information is stored.

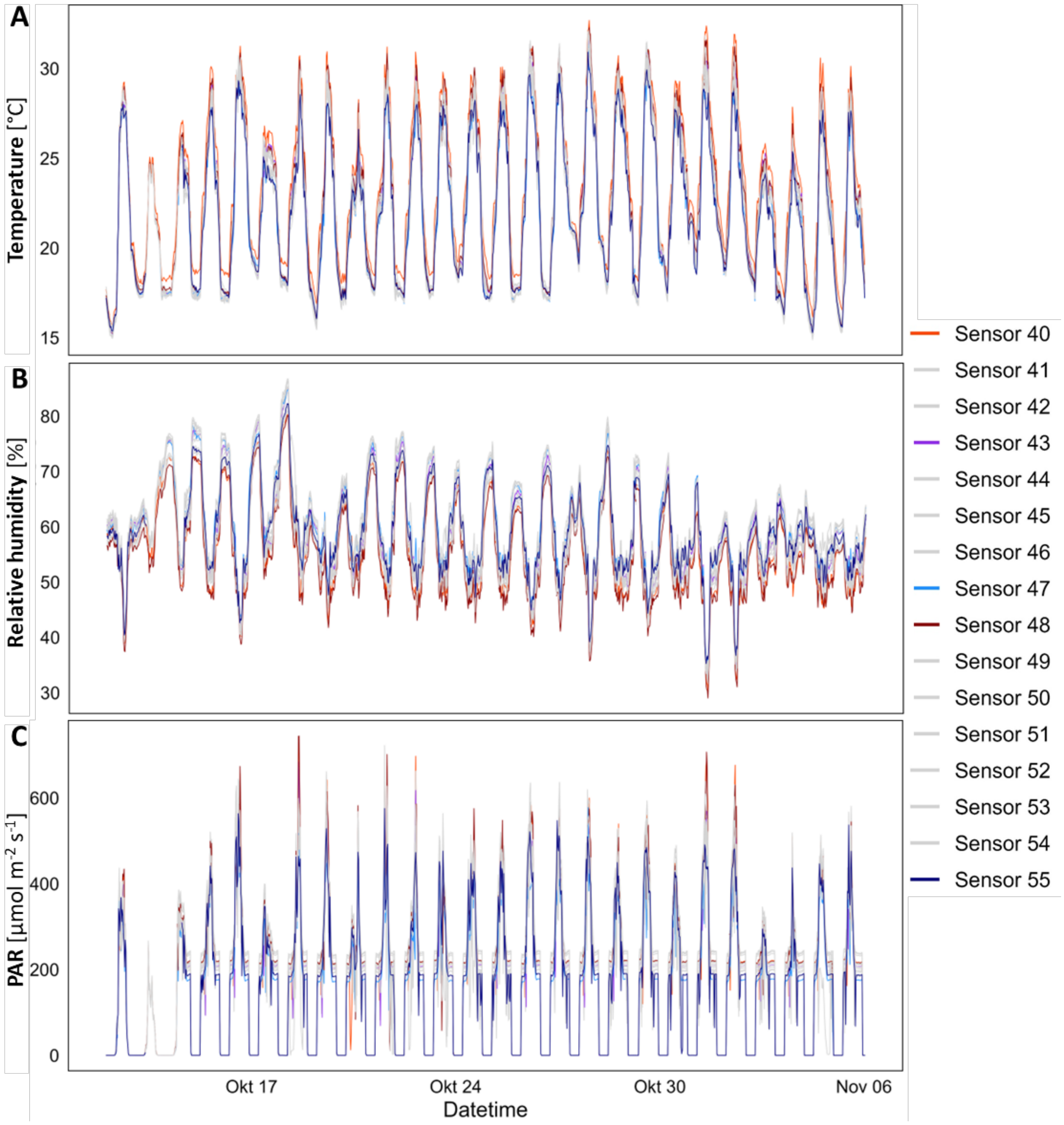

Fig. S6: Variation in microclimate within the plant cultivation area during the GrowScreen-Rhizo 3 experiment with 24 barley genotypes. A) Air temperature, B) relative air humidity, C) photosynthetically active radiation (PAR) recorded by thirteen sensors positioned on every second table.

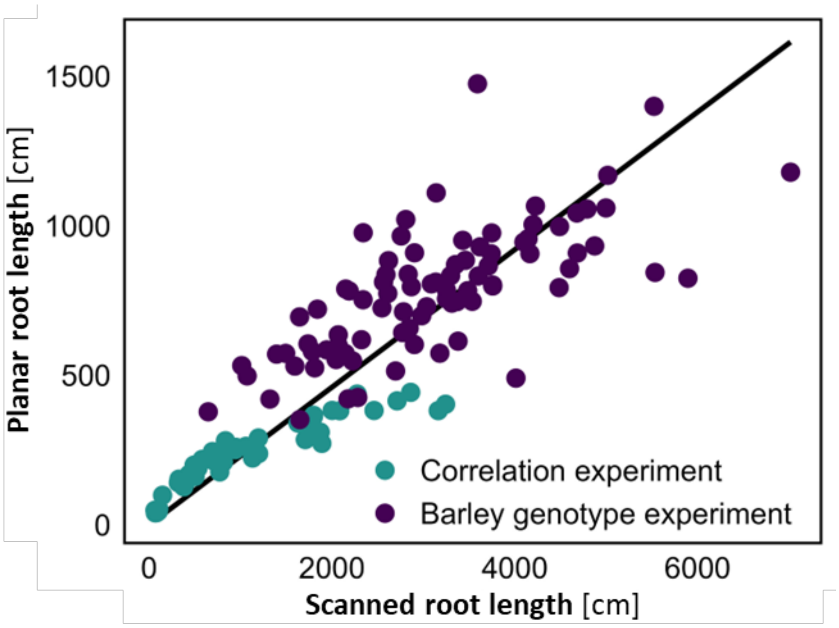

Fig. S7: Correlation between planar root length as derived from rhizotron images and scanned root length for two experiments for barley (first correlation experiment and genotype experiment). Regression line indicates significant linear fit (r^2=0.78).

Table S1: Definition of image-based and destructive traits gathered in GrowScreen-Rhizo 3 for barley plants. Italics indicate calculated traits.

Method	Trait	Unit	Trait definition
Daily automatic imaging of plants			
Shoot image analysis	Predicted leaf area ($LA_{predicted}$)	cm^2	Leaf area based on shoot images (for details see Material and Methods)
	Plant height	cm	Plant height based on shoot cameras (side view of shoots)
	Shoot growth rate	*cm^2 d^{-1}*	Growth rate based on predicted leaf area
Root image analysis	Root system depth	cm	Maximum vertical depth of a root system
	Root system width	cm	Maximum horizontal distribution of a root system
	Root system convex hull area	cm^2	Area of the convex hull that encompasses the whole root system
	Planar root length	cm	Total length of all visible roots (sum of seminal and lateral roots)
	Root system growth rate	*cm d^{-1}*	Growth rate based on planar root length
	Seminal root length	cm	Length of visible seminal roots
	Proportion of seminal roots	*cm cm^{-1}*	Proportion of seminal root length to planar root length
	Seminal root growth rate	*cm d^{-1}*	Growth rate based on seminal root length
	Lateral root length	cm	Length of visible lateral roots branched from seminal roots
	Proportion of lateral roots	*cm cm^{-1}*	Proportion of lateral root length to planar root length
	Lateral root growth rate	*cm d^{-1}*	Growth rate based on lateral root length
	Lateral to seminal root length ratio	*cm cm^{-1}*	Ratio of lateral to seminal root length
Combined image analysis	*Image-based root to shoot ratio*	*cm cm^{-2}*	Ratio of planar root length to predicted leaf area
Manual measurements at harvest			
Weighing	Shoot fresh weight	g	Fresh weight of total aboveground biomass
	Shoot dry weight	g	Dry weight of total aboveground biomass
	Root dry weight	g	Dry weight of root biomass
	Plant biomass	*g*	Sum of shoot and root dry weight
	Root to shoot ratio	*g g^{-1}*	Ratio of root and shoot dry weight
Manual assessment	Length of the longest leaf	cm	Length measured from rhizotron adapter to the tip of the longest leaf
	Tiller number	*[unitless]*	Manually counted number of tillers
Leaf area meter	Scanned leaf area	cm^2	Total leaf area determined by scanning all leaves after destructive harvest
Root scanning	Scanned root length	cm	Total root length determined by scanning all roots after washing
	Average root diameter	mm	Average root diameter of whole root system determined by scanning the washed roots
	Scanned root to shoot ratio	*cm cm^{-2}*	Ratio of total root length and leaf area after destructive harvest

Table S2: List of published soil-based root phenotyping platforms using clear pots or rhizotrons to quantify root traits in comparison to GrowScreen-Rhizo 3 platform.

Method/ Prototype name	Type of vessel/ dimensions	Substrate	Type of traits	Capacity/Typical experiment size	Imaging and resolution	Automation details	Sensor approach	Reference
**Controlled environment**								
Clear pots	Clear pots/ 4 L, 19 cm height	Pine bark potting substrate	Seminal root number and angles	24 seeds per pot, 600 plants/m^2	RGB (16 MP)	No automation, manual pot rotation and image acquisition	Plant-to-sensor	Richard et al. 2015
GLO-Root	Rhizotrons/ 30(height)x15x0.2 cm	Peat based substrate	Root length, root system width and root angles	96 rhizotrons	CCD cameras and bandpass filters (2048x2048 px)	Gantry system for automated rhizotron movement and imaging	Plant-to-sensor	LaRue et al. 2022
RootBot	Thin plates/ 24(height)x24x0.2 cm	Different soils/ substrates; addition of PEG	Root growth rates	more than 50	RGB (24 MP)	Gantry system for automated plate movement and imaging	Plant-to-sensor	Ruppel et al. 2023
RhizoPot	Rhizotrons/ 34(height)x20x8.5 cm	Agricultural subsoil	Root growth rates, root diameters, root hairs	not reported	Flatbed scanner (1200 px, 4800 px for root hairs)	Automated scanning	Sensor-to-plant	Zhao et al. 2022
WinRoots	Culture cases / 55(height)x100x2 cm	Soil substrate and vermiculite mixture	Primary root length	up to 2160 seedlings, 40 per culture case	Roots: RGB (18 MP), Shoots: Hyperspectral	Manual imaging acquisition	Plant-to-sensor	Zhang et al. 2022
Root-HTP	Rhizotrons/ 113(height)x54x4.8 cm	Agricultural subsoil	Time course analyses of global and dynamic root traits	not reported	RGB (3466x5196 px)	Automated imaging, use of AGVs	Sensor-to-plant	Zhang et al. 2025
**Climatized greenhouse**								
RhizoTubes	RhizoTubes/ 50 cm height, 18 cm diameter	Substrate / roots separated by permeable membrane	Global RSA traits, root nodules in legumes, shoot digital traits	1200 cylindrical vessels	RGB (6MP)	Automated image acquisition and integration in automated platform using conveyors	Plant-to-sensor	Jeudy et al. 2016
Low cost root phenotyping system	Rhizotrons/ 50(height)x45x1 cm	Clay textured soil	Nodal root angles	500 rhizotrons	RGB (20.2 MP)	Manual image acquisition	Plant-to-sensor	Joshi et al. 2017
PhenoRoots	Rhizotrons/ 80(height)x50x2 cm	Potting substrate	Global RSA traits	200 rhizotrons	RGB (18 MP)	Manual image acquisition	Plant-to-sensor	Martins et al. 2019
Rhizo-pots	Rhizotrons/ 40(height)x35x25 cm	Peat based potting substrate	Growth rates, global RSA traits, shoot traits, manual measurements	not reported	RGB and NIR (750 nm filter)	Automated imaging and plant transport with a conveyor belt system	Plant-to-sensor	Shi et al. 2023
GrowScreen-Rhizo 1	Rhizotrons/ 90(height)x70x5 cm	Peat based potting substrate	Growth rates, global RSA traits, shoot traits, manual measurements	72 rhizotrons	Roots: Monocrome (16 MP), Shoots: RGB (5 MP) from side views	Automated movable imaging station and rhizotron pickup system	Sensor-to-plant	Nagel et al. 2012
GrowScreen-Rhizo 2	Rhizotrons/ 80(height)x40x5 cm	Peat based potting substrate	Growth rates, global RSA traits, manual measurements	80 rhizotrons	Roots: RGB (29 MP), Shoots: RGB (5 MP) from side views	Automated imaging and plant transport with a fixed conveyor belt system	Plant-to-sensor	Nabel et al. 2018
GrowScreen-Rhizo 3	Rhizotrons/ 80(height)x40x5 cm	Peat based potting substrate	Growth rates, global RSA traits, manual measurements	896 rhizotrons	Roots: Monocrome (29 MP), Shoots (5 MP) from different view angles	Automated imaging and plant transport with AGVs	Plant-to-sensor	Presented in this study
